# The impact of mobile payment on rice farmers’ purchasing behavior of new rice varieties: a case study of rice cultivation in Jiangxi Province

**DOI:** 10.3389/fnut.2026.1711283

**Published:** 2026-06-23

**Authors:** Zhenghua Zhang, Junhao Yang, Lun Hu

**Affiliations:** School of Economics and Management, Jiangxi Agricultural University, Nanchang, China

**Keywords:** excellent new varieties, Jiangxi Province, mobile payment, purchase unit price, quantity purchased, rice farmers

## Abstract

**Introduction:**

With the rapid development of information and communication technology, mobile payment is becoming increasingly popular in rural areas of China. Rice farmers are accelerating their shift from offline cash payments to online mobile payments for purchasing excellent new varieties.

**Methods:**

Based on the purchasing scale and level of excellent new rice varieties, this study uses microdata from thousands of households in 100 villages in Jiangxi Province, employing the Heckman two-stage model, propensity score matching method, and instrumental variable (IV) method to explore the impact of mobile payment on the purchasing behavior of rice farmers regarding excellent new rice varieties and its mechanisms. Additionally, it investigates the heterogeneity of the impact of mobile payment on the purchasing behavior of rice farmers regarding excellent new rice varieties. Furthermore, it discusses the differences in the effects of mobile payment across different excellent new rice varieties.

**Results and discussion:**

Mobile payment has a significant impact on the purchasing scale and level of excellent new rice varieties among rice farmers, and it has passed tests for endogeneity and robustness; mobile payment can enhance land leasing levels by promoting land circulation, reduce the probability of “zero rent” land transfers, alleviate formal credit constraints, release the consumption potential of rural residents, optimize their consumption structure mechanisms, and promote rice farmers’ purchase of excellent new varieties. The group effect of mobile payment on rice farmers’ purchase of excellent new varieties is more pronounced among men and those with a high school education, while the scale effect is more significant among the elderly population, and the effect is more evident among the younger population. At the same time, the characteristics of mobile payment for rice farmers in purchasing excellent new varieties are more pronounced in breeding varieties that are drought-resistant, waterlogging-resistant, lodging-resistant, insect-resistant, and disease-resistant. This study indicates that mobile payment provides important references and experiential insights in the action plans for inclusive finance, empowering agricultural e-commerce, and revitalizing the seed industry.

## 1 Introduction

High grain yield starts with good varieties. The seed industry is the “chip” of agriculture, playing an extremely important role in ensuring food security and improving agricultural productivity. The Central Committee of the Communist Party of China (CPC) and governments at all levels attach great importance to the development of the crop seed industry, implementing a series of strategic reforms and arrangements from “implementing the seed project” to “developing modern breeding” and then to “the industrialization of biological breeding and the cultivation of leading enterprises in the seed industry.” In 2021, the Ministry of Agriculture and Rural Affairs promulgated the “Seed Industry Revitalization Action Plan,” emphasizing that “seeds are the foundation of agricultural modernization, and therefore, the national seed industry must be developed, elevating seed source security to a strategic height related to national security, focusing on solving problems, addressing shortcomings, strengthening advantages, controlling risks, and achieving self-sufficiency and self-control in seed industry technology”; in 2022, Document No. 1 of the Central Committee proposed to “fully implement the Seed Industry Revitalization Action Plan”; and in 2023 and 2024, Document No. 1 of the Central Committee proposed to “accelerate the revitalization of the seed industry, improve the joint research and application cooperation mechanism, increase key core technologies for seed sources, and accelerate the breeding and promotion of urgently needed excellent varieties.” In 2025, Document No. 1 of the Central Committee proposed to “deeply implement the revitalization of the seed industry.” The role of major agricultural scientific research platforms such as “Nanfan Silicon Valley” shall be fully exerted, breakthrough crop varieties shall be rapidly developed, and the national strategy for seed industry revitalization shall be comprehensively implemented.

After years of attention from the CPC Central Committee and the arduous construction of the breeding system, China’s seed industry has achieved good results. According to statistics, the total scale of China’s seed market in 2021 was approximately 121.41 billion yuan. According to the report of China Commercial Industry Research Institute, it is estimated that by 2025, China’s seed market will reach 148.067 billion yuan. The coverage rate of improved crop varieties in China exceeds 96%, and the varieties of major agricultural products have been replaced more than six times, with the area of self-selected varieties accounting for more than 95% (1). Among them, the varieties of rice and wheat are completely self-sufficient, with improved varieties contributing over 45% to grain production, and new varieties contributing 30% to the growth of agricultural production (2). For example, Jiangxi Province is one of the largest rice breeding provinces in China and has made significant contributions to the cultivation of new rice varieties. According to the “2023 Notice on Major Agricultural Varieties and Key Promotion Technologies” issued by the Jiangxi Provincial Department of Agriculture and Rural Affairs, it is pointed out that 22 new rice varieties will be cultivated in Jiangxi Province in 2023. By 2025, 14 new varieties, including Bingliangyou 309, Anyou 5,020, and Qiliangyou 2,216, were added in Jiangxi Province. Compared to traditional rice varieties, the new varieties are characterized by high yield, high quality, and good resistance, belonging to green, high-quality and high-yield rice varieties. The increase in new rice varieties has greatly enriched the supply of seeds and accelerated the promotion and application of rice production technology.

With the comprehensive promotion of excellent rice varieties, farmers have become the true decision-makers in the seed market, possessing complete independent choice in seed selection. However, in reality, farmers face the following issues when purchasing new rice varieties: First, farmers lack smooth channels to obtain comprehensive, accurate, and timely information on new rice varieties. Second, there is a lack of sufficient after-sales service guarantees; the supported cultivation techniques cannot be taught, technical guidance is not tracked, and the characteristics of new varieties cannot be fully utilized, resulting in final yields and benefits that are below expectations. Third, the single purchasing channels for seeds and the blind selection of new varieties lead to a reduction in the production of high-quality rice. Fourth, farmers themselves lack enough cultural knowledge, which prevents them from matching excellent varieties, good practices, and skills. The controversies arising from improper variety selection and the limitations of farmers’ own knowledge levels have put them in a dilemma regarding the selection of improved varieties, resulting in a low probability of purchase. Therefore, how to give play to the market mechanisms to guide the allocation of seed resources through farmers’ choices to purchase excellent varieties has become a focus of academic attention.

Some scholars believe that farmers’ purchase of excellent new varieties is influenced by internal factors (such as social experience, economic conditions, information media, education level, income sources, labor conditions, arable land area, and income level) and external factors (new variety brands, prices, characteristics, number of distribution points, and advertising) (3–6). In addition to the above factors, mobile payment has become another important factor affecting rice farmers’ purchase of excellent new varieties. The widespread popularity of smartphones, the opening of fast payment interfaces by banks, the rapid development of mobile Internet technology, and the extensive application of QR code scanning technology and WIFI have solved the problem of the dual hardware transformation of mobile payment terminals through software. The entry barriers to the mobile payment market have basically been eliminated, and it has gone through four stages of development. In the first stage, the demand for convenient payment methods increasingly promoted the birth of mobile payment; in the second stage, the gradual improvement of functions and market expansion is characterized by the initial transition from basic functions of transferring and receiving payments to paying for living expenses such as telephone bills, gas bills, and water bills. With the expansion of the market for power and wealth management products, the third stage is the growth stage of a diversified payment ecosystem. On the one hand, it continuously enriches payment scenarios, including common consumption scenarios such as shopping, catering, and travel, and extends to public service fields such as healthcare, education, and government affairs, achieving comprehensive coverage of daily life. On the other hand, a series of value-added services, such as wealth management, and financial products like changes, have been launched to provide users with channels for capital appreciation. The fourth stage, the global influence stage, is characterized by the expansion of mobile payment from the domestic market to the international market. On 29 August 2024, the China Internet Network Information Center (CNNIC) released the “54th Statistical Report on Internet Development in China,” which showed that as of June, the online payment utilization rate among Internet users aged 60 and above in China had reached 75.4%. In the first half of the year, more than 5 million immigrants used mobile payment, a 4-fold increase year-on-year.

Under this development background, mobile payment has reached the “last mile” in rural areas, narrowing the “digital divide” between urban and rural areas. With the advantages of mobile Internet technology, wide coverage, convenient operation, and efficient service, mobile payment has improved circulation efficiency, increased farmers’ income, and supported rural revitalization in areas such as agricultural material distribution, agricultural product circulation, and supply chain connections. According to the “47th Statistical Report on Internet Development in China” released by CNNIC, by the end of 2020, the mobile payment utilization rate in rural areas is expected to reach 79%. By the end of 2024, the penetration rate of mobile payment in rural areas of China is projected to reach 93.8%, with the number of users reaching 960 million. The ecological environment for rural payments is developing healthily. Since 2024, the penetration rate of mobile payment in rural areas of Henan Province has reached nearly 80%, and the cumulative number of users of the “China UnionPay QuickPass” APP has reached 15.7432 million, an increase of 8.71% since the beginning of the year. Mobile payment is deeply integrated with agricultural product sales channels, which promotes the digital upgrade of agricultural products exhibition and purchase from offline to online markets. Farmers are proficient in using mobile payment tools, such as mobile banking and China UnionPay QuickPass, to ensure that “money is in hand, and seeds are sown.”

To fill the gaps in previous research, this study employs the Theory of Rational Behavior and the Theory of Consumer Value, using the Heckman two-stage model to explore the impact of mobile payment on the purchasing behavior of rice farmers regarding excellent new varieties. Furthermore, the robustness of the conclusions is verified using alternative sample values, alternative independent variables, tail shrinkage treatment, and alternative model methods. At the same time, the propensity score matching method combined with IV methods studies the endogeneity issue of mobile payment on the purchasing behavior of rice farmers regarding excellent new varieties, further exploring the relational mechanisms and individual characteristic heterogeneity, and finally analyzing the characteristic differences in rice farmers’ purchases of excellent new varieties. Finally, it explores policy suggestions for farmers’ choices to purchase excellent varieties in the context of mobile payment and the role of market mechanisms in guiding seed resource allocation.

The reason for this study is “The country is agriculture-oriented, and agriculture prioritizes species.” In agricultural production, seeds are vital special production materials that can improve crop yield and product quality, becoming the core application of agricultural production technology measures and an important carrier and manifestation of agricultural scientific and technological progress. Due to farmers’ persistent pursuit of high-yield and efficient production goals, the demand for new varieties is also very strong. Mobile payment plays a significant role in promoting the scale and level of rice farmers’ purchases of excellent new varieties, and by facilitating the transfer of agricultural land, it can improve the rent level of agricultural land, reduce the probability of “zero rent” transfers, alleviate formal credit constraints, release the consumption potential of rural residents, optimize their consumption structure mechanisms, and promote rice farmers’ purchases of excellent new varieties. This promoting effect needs to be observed across different genders, education levels, and age structures. At the same time, it is necessary to further investigate the characteristic differences of different new varieties. This study solves this problem on the basis of the above research. As an infrastructure, mobile payment can help policies accurately reach rice farmers, promote the large-scale planting of high-quality varieties, and assist in rural revitalization, which aligns with the development policy orientation of high-quality food projects repeatedly emphasized in Document No. 1 of the Central Committee.

The significance of this study lies in the following: First, mobile payment can increase the number of excellent rice varieties purchased by rice farmers, promote the promotion and popularization of excellent rice varieties, help farmers scientifically select suitable varieties, and promote high-quality and tasty rice. Second, mobile payment promotes rice farmers’ purchases of high-quality rice by reducing the probability of “zero rent” farmland transfers, which is conducive to rural land transfers and large-scale planting. Mobile payment promotes rice farmers’ purchases of high-quality rice by relaxing formal credit restrictions, helping to convert mobile payment data into “credit capital,” assisting rice farmers in establishing credit profiles, and facilitating access to bank loans or agricultural insurance support. Mobile payment effectively promotes this by releasing the consumption potential of rural residents and optimizing their consumption structure. Third, there are differences in the promotion of mobile payment for rice farmers’ purchases of excellent new varieties across different genders, ages, and educational levels, indicating that policymakers need to examine the differences among different groups when promoting new rice products. Fourth, the characteristics of mobile payment in promoting rice farmers’ purchases of excellent new varieties are more pronounced in drought-resistant, waterlogging-resistant, lodging-resistant, insect-resistant, and disease-resistant breeding varieties. This shows that the high-yield, high-quality, and multi-resistant new varieties bred by breeders are quickly applied in production, which is not only an effective management approach to increase yield, reduce investment, and increase profits but also has significant implications for controlling diseases, reducing the use of fertilizers and pesticides, protecting the environment, and promoting the production of green food.

The purpose of this study is to explore the impact of mobile payment on rice farmers’ behavior in purchasing excellent rice varieties. It studies its endogeneity, mechanisms, and heterogeneity. It promotes the expansion of purchasing channels for high-quality rice, facilitates land transfer to realize large-scale planting, optimizes capital management and the circulation of credit support information, upgrades the structure of agricultural consumption, and attaches importance to the role of excellent rice variety characteristics in the production of quality grains.

To sum up, as an important part of digital finance, current research on mobile payment mainly analyzes its impact on family financial behavior from aspects such as entrepreneurship, consumption, and money demand (7, 8). However, there is still a lack of research literature that combines mobile payment with rice farmers’ purchase of excellent new varieties at home and abroad. Under this background, this study uses survey data from 100 micro-farmers in villages in Jiangxi Province to explore the heterogeneity and mechanisms of the influence of mobile payment on the scale and level of rice farmers’ purchases of excellent new varieties. The contributions of this study include the following: First, based on the rapid development of the mobile payment market in China and the narrow channels for rice farmers to purchase excellent new varieties, it systematically studies the impact of mobile payment on the scale and level of rice farmers’ purchases of excellent new varieties, expanding and enriching the research in the field of difficult-to-sell and purchase new varieties; second, it identifies and verifies the channels through which mobile payment affects rice farmers’ purchasing behavior for excellent new varieties; third, through heterogeneity analysis, it further studies the heterogeneous effects of family endowments and the characteristics of new rice varieties. The above research provides a useful perspective and experience to partially explain the mystery of “difficult to sell and difficult to purchase” in China’s agricultural product circulation market, improves the market construction of the rural seed industry, and achieves the goal of rural revitalization.

## 2 Case analysis of seed purchasing behavior and food quality, dietary results, and nutritional effects of food system

### 2.1 A typical case of rural farmers in China

Ordinary farmers in the main grain-producing areas of central China can be divided into two groups: One gives priority to purchasing high-yield hybrid seeds, pursuing yield and sales income; the other tends to buy disease-resistant ecological varieties, nutritious mixed grain varieties, and local old varieties, taking into account the eating quality and family dietary health. The agricultural department uniformly guides farmers to purchase high-quality and nutrient-rich coarse grain seeds and standardizes seed purchasing channels and variety selection. The coarse grains planted contain high levels of nutrients such as protein, dietary fiber, and anthocyanins, and all food safety indicators meet standards. On the one hand, high-quality mixed grains enter the dining table, improving the staple food structure of residents, reducing excessive intake of polished rice and white flour, and effectively addressing dietary health issues such as “three highs” (high blood pressure, high blood sugar, and high cholesterol) and gastrointestinal discomfort; on the other hand, high-quality mixed grains form characteristic agricultural products flowing into the market, enriching the categories of dietary components in society, optimizing the nutritional supply layout of the national food system, and realizing farmers’ purchasing behavior, directly linking public nutritional health with the nutritional upgrade of the food industry.

### 2.2 The relationship between seed purchasing behavior and food quality, dietary results, and nutritional effects of food system

#### 2.2.1 The correlation between seed purchasing behavior and food quality

Farmers who only purchase high-yield economic seeds have weaker resistance and are easily affected by pests and diseases, frequently spraying pesticides during the planting process, resulting in high pesticide residues in agricultural products, poor fruit taste, and low vitamin and mineral content; blindly purchasing low-priced bulk inferior seeds leads to uneven seedling emergence, crops being easily affected by diseases, poor appearance of agricultural products, increased probability of mold, and poor basic food quality. Farmers who purchase conventional high-quality improved varieties and ecological disease-resistant seeds experience fewer pests and diseases in the field, streamlined agricultural inputs, compliant pesticide residues, low heavy metal accumulation, uniform maturity of components, and complete retention of original nutritional components, significantly improving food safety and physical quality.

#### 2.2.2 The correlation between food quality and dietary results

Families of farmers who consume low-quality, high-yield agricultural products have a monotonous diet with insufficient nutritional density. Their staple foods and vegetables only meet the need for satiety, leading to inadequate intake of dietary fiber and trace elements, which can easily result in physical weakness and low immunity. The diet only fulfills the function of satiety and fails to achieve the goal of a healthy diet. In contrast, families that consume high-quality, organic agricultural products enjoy authentic flavors and rich nutrition, with a more reasonable dietary structure. They combine local grains, fruits, and vegetables, significantly enhancing dietary comfort and health experience.

#### 2.2.3 The association between dietary results and nutritional safety

Long-term consumption of low-quality, single-component diets can lead to issues such as hidden hunger, mild malnutrition, anemia, and developmental delays among rural, left-behind elderly and children, resulting in weak nutritional security at the family level; health components produced from high-quality seeds can fill nutritional gaps in daily diets, reduce the risk of chronic diseases caused by diet, and stabilize basic nutritional security for families.

#### 2.2.4 Extending nutritional effects to food systems

Farmers in the region generally follow trends to purchase high-yield grain seeds, leading to a highly homogenized local planting structure, with a sharp decline in the planting area of miscellaneous grains and high-nutrient crops, resulting in a food supply that trends toward high-calorie, low-nutrition options, and an imbalanced nutritional supply structure in local food systems; some villages collectively promote high-quality nutritional seeds, restore local germplasm cultivation, diversify regional food supply, improve the overall nutritional supply capacity of county-level food systems, and enhance the health protection level of people’s lives.

### 2.3 Summary

From practical cases, seed purchasing behavior is the starting point of the entire nutrition and food chain. The types of seeds chosen by farmers, the channels through which they purchase seeds, and their seed preferences directly determine the food quality of agricultural products, thereby altering the daily dietary structure and health results of residents. This not only affects the micro-nutritional security of families but also adjusts the regional planting structure and food supply structure after long-term agglomeration, ultimately profoundly impacting the nutritional supply efficiency, supply diversity of the entire food system, and the overall effects on national nutrition and health.

## 3 Theoretical analysis and hypotheses

### 3.1 Definition of excellent Rice varieties

The definition of excellent new grain food varieties by the Chinese municipal government is as follows: By 2025, the new excellent rice varieties promoted by the state will include four types: backbone, growth, budding, and specialized.

Table 1 introduces the list of excellent new varieties recommended by the country, along with the publication year threshold and shape standards, as shown in Table 1.

**Table 1:** Promotion of national new rice varieties in 2025.

New variety type	List of national recommended rice varieties	Year threshold	Shape standard
Backbone variety	Jingliangyou 534, Yexiang Youlisi, Quanyou 822, Yixiangyou 2,115, Huang Huazhan, Longjing 31, Suijing 27, Nanjing 9,108, Zhongjiazao 17, Zhongzao 39, and Jingliangyou Huazhan.	Approval (registration) promotion for 5 years or more.	The main varieties of grain, cotton, oil, and sugar entered the top 10 in the suitable ecological area for 3 consecutive years, and the varieties of fruit, vegetable, and tea entered the top 10 in China for 3 consecutive years.
Growing variety	Quanyou 1,606, Bangliangyou Yuxiang, Changliangyou No. 8, Weiliangyou 8,612, Huazheyou 210, Chuanyou 6,203, Nanjing 5,718, Jijing 830, Zhenliangyou 8,612, Taiyou 808, Weiliangyou 7,713, Ningxiangjing No. 9, and Yongyou 1,540.	Approval (registration) promotion for 3 years or more.	Outstanding performance in the national core exhibition base or provincial exhibition evaluation, the promotion area has increased rapidly, and it has entered the top 30 in the suitable ecological area (grain, cotton, oil, and sugar) or the whole country (fruit, vegetable, and tea) and is expected to grow into a backbone variety.
Emergent variety	Zhuyou Zhunong Silk Miao, Shaoxiang 100, Geliangyou 5,117, Changsheng Youzhen Silk Miao, Shengdao 259, Zhongzao 83, Quanyou E Fengsi Miao, Lianhui 6,612, Bangliang You Xiang Zhan, Anliangyou 2, Yangxian You 4,278, and Weiliangyou 2,268.	Approval (registration) promotion is less than 3 years.	The yield, resistance, and quality are all good, and the comprehensive characters are excellent. It has excellent performance in the national core exhibition base or provincial exhibition and evaluation, and has great market potential. The formation enterprise or the integrated enterprise of breeding, breeding, and promotion is expected to become a growing and backbone variety.
Specially specialized variety	Qingliangyou 3,261 (salt-tolerant rice), Hanyou 73 (water-saving and drought-tolerant rice), and Yongyou 4,949 (ratooning rice).	Newly approved (registered)	Special excellent new varieties (such as saline-alkali tolerant varieties) that meet the consumption needs of diversified markets and can significantly improve the utilization rate of resources such as land, fertilizer, water, light and temperature, or varieties that have made breakthroughs in yield, resistance, quality, growth period, mechanization, and new farming system (such as ratooning rice, strip compound planting, and short growth period rape).

### 3.2 Theoretical analysis

The rational choice theory assumes that farmers make rational decisions when selecting new rice varieties to ensure maximum utility. According to this theory, the choice of new rice varieties is influenced by both supply and demand. For consumer farmers, Solomon (9) and Estefan A (10) think that the factors influencing farmers’ purchase of new rice varieties can be summarized as personal characteristic variables, including age, sex, education level, and whether they have joined a cooperative; income structure variables, including rice production area, sales volume, production income, and rice yield; and cognitive variables, including the effects of yield increased production, seed characteristics, and price, as well as familiarity with the seeds. Rutsaert (11) thinks that the variables affecting the environment (such as soil characteristics, planting experience, the influence of friends and neighbors, recommendations from agricultural technicians, promotions by seed companies and the government, the convenience of transportation facilities, and media publicity) play a significant role. For suppliers, seed performance, brand, quality, growth, price, suggestions from retailers, demonstrations, and farmer meetings are the most effective means of marketing rice seeds (12).

In recent years, scholars have focused on the impact of mobile payments on rural production, consumption, marketing activities, and economic transformation. On the one hand, in terms of consumption, Wei (13) believed that the application of mobile payment has significantly promoted the household consumption of rural residents, with an increase ranging from 29.8 to 52.3%. This has been mainly achieved by alleviating the liquidity constraints of farmers, enriching their consumption choices, and improving the convenience of payment for farmers; on the other hand, in terms of production, Mwikamba (14) found that using mobile phones can promote the production efficiency and productivity of climate-smart horticultural farmers. Cai (15) found that the use of smartphones has a positive impact on the willingness to adopt digital pest and disease management. Kolapo (16) also reached the same conclusion. In addition, in marketing agricultural products, Brighton (17) believes that the use of smartphones by small-scale rural farmers in Zimbabwe has a positive impact on their willingness to access and adopt agricultural market information. Finally, in terms of rural economic transformation, Min (18) stated that the use of smartphones by farmers has a significant impact on rural economic transformation, promoting non-agricultural employment, the cultivation of non-food crops, and the specialization of crops among family members.

In view of this, mobile payments provide a solid theoretical foundation for promoting rural production, consumption, marketing activities, and economic transformation, but there is still room for research and development. The lack of systematic investigation into the impact of mobile payments on farmers’ purchasing behavior for quality varieties limits the influence of Internet economy development and rural agricultural products sales. Therefore, based on survey data from micro-farmers in China’s grain planting areas, this study explores the influence of mobile payments on farmers’ purchasing behavior for quality varieties and further investigates its mechanisms, heterogeneity, and the characteristic differences of different quality varieties, which is helpful to formulate measures to correct farmers’ purchasing biases for quality varieties from a differentiated perspective.

### 3.3 Research hypotheses

#### 3.3.1 The influence of mobile payments on the purchasing behavior of excellent new varieties of rice farmers

The promotion of mobile payment for rice farmers to purchase excellent new varieties mainly reflects the following two aspects: first, the trust mechanism. With the development of technologies such as artificial intelligence, mobile payment empowers traditional production factors, constructing a transparent, secure, and efficient trust mechanism to ensure payment safety and personal privacy, effectively alleviating the problem of information asymmetry (19). Third-party payment platforms, such as Alipay and WeChat, have built an intermediary platform for rice farmers, establishing a credit transfer mechanism, expanding the narrow social trust radius determined by blood ties, geography, and kinship, and promoting farmers to use transfer payments to purchase improved varieties. Wang Yangjie et al. pointed out that in the modern transaction system, mobile payment can reduce the sale of inferior and counterfeit rice varieties to protect the legitimate interests of rice farmers. After multiple online market transactions, rice farmers have formed stable trust in third-party payment platforms and are more inclined to use mobile payment to purchase high-quality varieties (20). Second, the information acquisition mechanism. Mobile payment has increased rice farmers’ attention to information about excellent new varieties and reduced their cost of obtaining information. On the one hand, as an important part of the new digital economy, mobile payment relies on Internet and cloud computing technologies, providing families with a safe and reliable channel to obtain information about excellent new varieties, thereby reducing the cost of information acquisition (21). For example, the promotion of agricultural information through Alipay, WeChat, and mobile banking can invisibly increase the attention of rice farmers, allowing them to obtain information about excellent new varieties from home; on the other hand, as a carrier of information transmission functions, WeChat effectively breaks the limitations of physical space, expanding the social network of rice farmers and connecting different groups of rice farmers through the function of information interaction. Moreover, the network relationship among rice farmer members is an important channel for them to obtain information about excellent new varieties. Network members can form WeChat groups to constantly exchange and transmit information about new varieties with great breeding value, such as cold tolerance, drought resistance, salt and alkali tolerance, disease resistance, and new herbicide resistance and promote farmers to purchase high-value new varieties through mobile payment. Based on the above reasons, this study puts forward the following hypothesis:

*H1*: Mobile payment can significantly promote the scale and level of rice farmers’ purchase of excellent new varieties.

#### 3.3.2 Mechanism for promoting market-oriented transformation transfer of agricultural land

The non-market nature of agricultural land transfer is due to the closed nature of rural primitive society and the inefficient order characteristics of the objects of agricultural land circulation. Mobile payment products embedded in e-commerce platforms, applications from commercial banks, and the service mode of Huinong APP can not only effectively alleviate transfer restrictions but also improve payment convenience and operational efficiency, promoting the flow of production factors between urban and rural areas and industries, breaking the inefficient order model of agricultural land transfer objects.

Mobile payment integrates new generation information technologies such as the Internet of Things, big data, blockchain, and artificial intelligence, promoting the two-way flow of production factors between urban and rural areas, with an increasing range and speed. The activity radius and network circle of rice farmers are constantly expanding, breaking the circle of acquaintances (11). Rice farmers’ foreign exchange interactions have become more frequent, while interactions and reciprocal relationships among neighbors are decreasing. The originally closed villages are further opening up, weakening the foundation of relationship governance, and gradually enhancing market-oriented transactions and profit motives. Under the background of the rapid development of mobile payment, the pattern of differentiated rural order is being dismantled, shaking the foundation of human society, which promotes the transition from a “relationship-based” agricultural land circulation market to a “contract-based” market. In short, with the help of mobile payment financial services and transaction services, traditional acquaintance-based land transfer transactions are being broken, providing rice farmers with more opportunities to communicate with the outside world, encouraging them to view market profits as the main subject of land circulation and actively seek larger market transaction counterparts (22). Therefore, the income that rice farmers gain from the marketization of agricultural land transfer is higher than that from non-marketized income, and farmers with higher incomes are more willing to purchase excellent new varieties. Based on this, the following hypothesis is proposed:

*H2*: By promoting the market-oriented transformation of farmland circulation, mobile payment can increase the level of farmland rent and reduce the probability of “zero rent” farmland circulation, thereby enhancing the scale and level of purchasing excellent new varieties.

#### 3.3.3 Mechanism for relieving credit constraints

Rice farmers are the demand side for excellent rice varieties. In China, small farmers, as the main force in agricultural production, are constrained by low agricultural returns and a lack of knowledge. Small farmers lack the willingness and ability to expand production and invest in new products; they tend to seek old, cheap rice seeds to maintain agricultural food production. Suppliers of excellent new varieties are mainly new variety sellers and banks that provide a large number of varieties. However, most excellent new varieties are expensive, and rice farmers cannot obtain high-quality varieties through offline sellers. In addition, due to credit exclusion caused by a lack of funds and information asymmetry with formal financial institutions, rice farmers are unable to access excellent new varieties under dual constraints (23). Therefore, the limitations of supply and demand prevent rice farmers from purchasing excellent new varieties.

Mobile payment can alleviate the above difficulties by easing credit constraints and improving the availability of financial services. On the one hand, mobile payment can quickly and conveniently link related online credit services, with WeChat providing small loans and micro-loan services, while Alipay offers small loan services, significantly alleviating the liquidity constraints faced by rice farmers, enabling their economic activities, which were previously limited in purchasing excellent new varieties, to receive financial support and promote the purchase of high-quality varieties (24); on the other hand, as a financial service tool, mobile payment helps banks understand the credit status of rice farmers, assisting them in overcoming the dilemma of being unable to obtain formal credit due to inaccurate information, effectively alleviating the liquidity constraints for rice farmers in purchasing excellent new varieties. Based on this, the following hypothesis is put forward:

*H3:* Mobile payment can improve the scale and level of purchasing excellent new varieties by alleviating formal credit constraints.

#### 3.3.4 Rural residents’ consumption potential release and consumption structure mechanism optimization

Rice farmers purchase excellent new varieties from traditional farmers’ markets and fairs, and their shopping channels are limited by time and space, thus restricting the consumption demand for excellent new varieties. However, the “information into the village” project and the digital rural strategy have accelerated the extension of new forms such as e-commerce and online retail into rural areas (25). The consumption channels for rice farmers to purchase excellent new varieties have expanded from offline to online, making the channels for purchasing excellent new varieties more diverse and convenient. Mobile payment, as an important information medium, has made rural areas no longer an “island” of consumption information (26). It provided comprehensive product information on high-quality varieties for rice farmers, which to some extent released the consumption potential of rice farmers to purchase high-quality varieties. The mobile Internet can timely and accurately push promotional information for high-quality products and conduct targeted push and display through search memory and big data analysis functions, improving the matching efficiency between high-quality product information and rice farmers’ demand for quality and low prices, increasing the flexibility of obtaining and consuming high-quality product information (27). To some extent, it promotes them to purchase more diversified and higher-value quality purebred products within the same budget. In addition, the improvement of logistics distribution and online after-sales service provides after-sales guarantees for rice farmers purchasing excellent new varieties, continuously upgrading the consumption structure of excellent varieties at high prices. Based on this, the following hypothesis is proposed:

*H4*: Mobile payment can release the consumption potential of rural residents, optimizing their consumption structure, thereby increasing the scale and level of purchasing excellent new varieties.

## 4 Data source, variable selection, and model setting

### 4.1 Data sources

**Figure 1:**
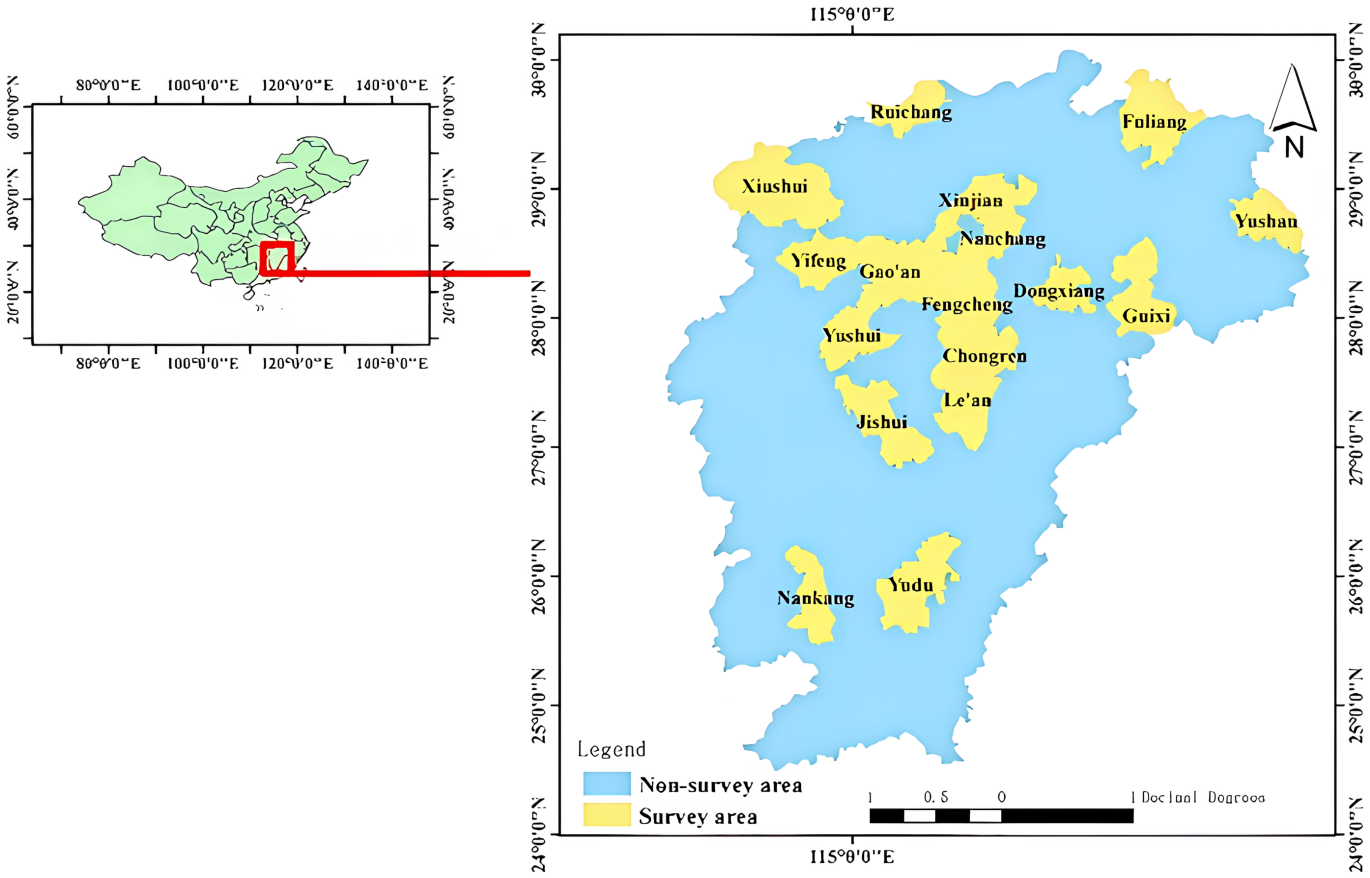
Survey area map.

The data in this study come from the survey database of “100 villages and 1,000 households” in Jiangxi Province in 2022, jointly launched by Jiangxi Agricultural University and Peking University. The sample was obtained through stratified random sampling. The survey area is shown in Figure 1. According to the per capita industrial added value of county-level administrative regions in Jiangxi Province, it is divided into 12 districts, with one sample county-level administrative unit randomly selected from each district. Each sample county-level administrative unit is divided into three equal parts according to the per capita public financial revenue, from which one sample township unit is randomly selected; each sample township unit is divided into three equal parts based on terrain and regional distribution, from which one sample administrative village is randomly selected; from each sample administrative village, 10 sample households are randomly selected for the survey. The survey covers various aspects, such as farmers’ characteristics, mobile payment, rice planting, seed selection, financial loans, consumption expenditure, and non-agricultural employment, and the sample is well-representative. At present, data from 1,080 farmers in 108 administrative villages have been collected. Among the surveyed area, 462 farmers are non-agricultural workers who do not plant high-quality rice or purchase high-quality rice. Therefore, these 462 farmers were excluded. The sample used in this study consists of 618 rice-growing farmers. After excluding abnormal values and missing values, the final effective sample is 537, with a sample validity rate of 86.89%. The survey sample covers data on farmers’ characteristics, mobile payment, rice planting, seed selection, financial loans, and consumption expenditure.

**Figure 2:**
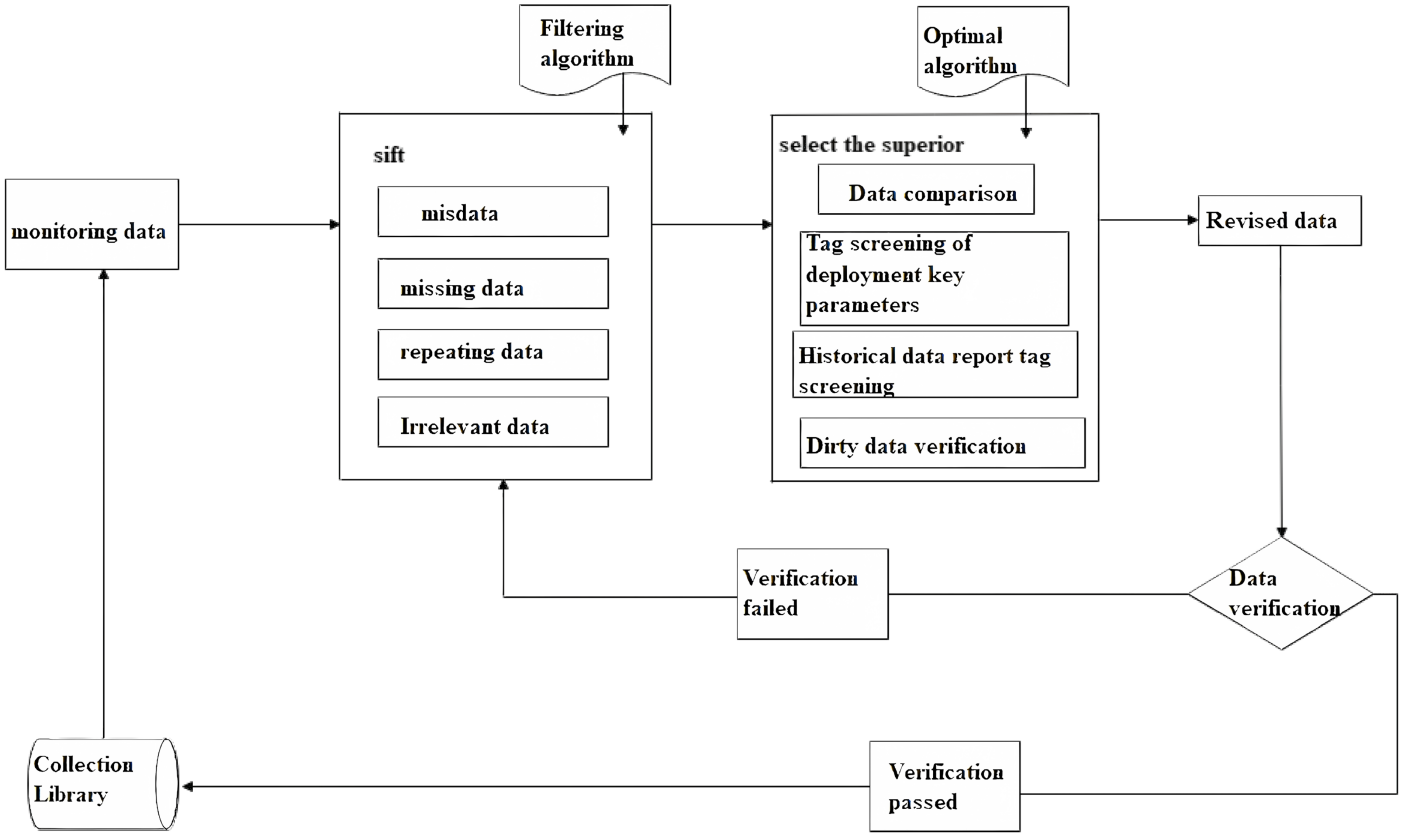
The cleaning flowchart.

A total of 1,080 data points were distributed in this study, with 618 being rice planting samples. The main reason is that the 462 farmers in the sample survey area are non-agricultural households. What we mean is that they are non-agricultural workers and do not plant agricultural high-quality rice, let alone purchase high-quality rice. Therefore, the sample used consists of 618 farmers who are engaged in agricultural work. The flowchart for data cleaning is a graphical tool that represents the steps of data cleaning. As shown in Figure 2, it usually includes the following steps: First, the raw data are collected and imported; then, the data are preprocessed, including removing duplicates and filling in missing values. Next, the data are transformed, such as converting the character strings to numerical data. After that, the data are normalized to meet analysis requirements; finally, the cleaned data are verified and evaluated. Throughout the process, multiple iterations and adjustments may be needed to ensure that the data quality meets the expected standards. In a word, the flowchart for data cleaning is an effective method that can help us better understand and master the process of data cleaning. The flowchart is as follows:

### 4.2 Variable selection

1. Dependent variable. In this study, whether farmers purchase excellent new varieties, the quantity of excellent new varieties, and the unit price are selected as dependent variables. Among them, the situation where farmers purchase excellent new varieties is assigned a value of 1 and a value of 0. Table 2 reflects the high-quality characteristics of the excellent variety Jingliangyou 534 in the survey sample, as shown below:
2. Core explanatory variables. In China, mobile payment methods when shopping include cash, credit cards (including bank cards), payment via computer (including online banking and Alipay), payment through mobile terminals such as mobile phones and iPad (including Alipay APP, WeChat Pay, mobile banking, and Apple Pay), and others. In this study, referring to Leng (28), combined with the questionnaire, farmers will use at least one of the following as a mobile payment tool: “direct transfer through bank accounts or other types of formal financial institutions’ accounts, payment through bank cards, payment through mobile WeChat or Alipay, or payment through online banking or online platforms,” and at least one will be assigned a value of 1; otherwise, it will be assigned a value of 0.
3. Control variables. Various control variables are included in this study to improve the accuracy of the model. These variables can reduce the risk of estimation errors caused by missing factors. Among them, family characteristics include gender, years of education, age, health status, and marital status; family characteristics include family population and arable land area; planting and production characteristics include rice yield, grain storage time, whether herbicides are used, whether pesticides are applied, and whether manual pesticides are used. The characteristics of excellent new varieties include drought resistance, waterlogging resistance, lodging resistance, insect resistance, and disease resistance (For details, see Table 1.).

**Table 2:** Jingliangyou 534 characteristics.

Variety characteristics	Good fertility, strong regeneration ability, good taste, and resistance to rice blast.
Examination and approval number	Guo Shendao 2016605, Guo Shendao 20176,004, Guo Shendao 20180044, Guo Shendao 20190033, Gui Shendao 2018037, Min Shendao 20180015, and Gui Shendao 2020202.
Variety right number	CNA20151615.4
Breeder	Yuan Longping Agricultural Hi-Tech Co., Ltd., Guangdong Academy of Agricultural Sciences Rice Research Institute, Shenzhen Longping Jingu Seed Industry Co., Ltd., and Hunan Longping Hi-Tech Seed Industry Science Research Institute Co., Ltd.
Variety source	Crystal 4155S × R534
Characteristics	Indica two-line rice varieties. Planting mid-season rice in the middle and lower
	reaches of the Yangtze River, the whole growth period is 136–137 days. The plant height is 118–122 cm, the ear length is 25. 1–25.6 cm, the total number of grains per ear is 196.1–204.9, the seed setting rate is 83.5–88.3%, and the 1,000-grain weight is 23.8–24.0 g. The comprehensive index of rice blast is 1.7–2.1, the highest loss rate of ear neck blast is 1 grade, bacterial blight is 5 grade, and brown planthopper is 9 grade. The rice quality has reached Grade 2 of the national high-quality rice standard, with brown rice rate of 77.6–80.3%, head rice rate of 52.1–68.0%, chalkiness of 2.8–2.9%, transparency of 1.0, alkali extinction value of 4.8–5.6, gel consistency of 79.0–86.7 mm, and amylose of 15.3% In 2014, he participated in the regional test of late maturity group of medium indica rice in the middle and lower reaches of the Yangtze River, with an average yield of 630.2 kg per mu, which was 6.26% higher than that of the control variety. In 2015, the average yield per mu was 644.4 kg, which was 5.28% higher than that of the control variety. In the production test in 2015, the average yield per mu was 681.2 kg, which was 5.10% higher than that of the control varieties.
Key points of cultivation techniques	Mid-season rice planting in the middle and lower reaches of the Yangtze River. 1. Sow at the right time. Generally, seeds are sown from late April to early May, and the seeds are soaked with strong chlorine essence for disinfection before sowing. 2. Insert basic seedlings. The mechanical transplanting age is approximately 4 leaves, the plant spacing is 13.3 cm × 20.0 cm, and 2–3 rice seedlings are inserted into each hole. 3. Scientific management of fertilizer and water. Generally, 12 kilograms of pure nitrogen, 6 kilograms of phosphorus pentoxide, and 9 kilograms of potassium oxide are applied per mu. Conventional water management, water cut off about 7 days before harvest.
Suitable promotion area	It is suitable for planting mid-season rice in the Yangtze River valley rice areas in Jiangxi, Hunan, Hubei, Anhui, and Jiangsu, as well as in the north of Fujian and the south of Henan. It is suitable for planting one-season mid-season rice in middle and low altitude indica rice areas in Yunnan and Guizhou, indica rice areas below 800 meters in Chongqing, hilly rice areas in Pingba, Sichuan, and rice areas in southern Shaanxi. It is suitable for planting early rice and late rice in Guangdong (except the rice-growing areas in northern Guangdong), southern Guangxi, Hainan, and southern Fujian but not in the areas with severe bacterial blight.
Development and promotion unit	Hu’ nan Longping seed industry co., Ltd.
Promotion situation	12.87 million mu (2021–2023).

As can be seen from Table 3, 83.9% of farmers purchased excellent new varieties, with an average purchase quantity of 26.450 kg and an average of 32.699 kg. In total, 41.9% of farmers use mobile payments. Men account for 79.1%, with an average of 6.495 years of education, and the average age of respondents is 59.23 years. The sample shows that farmers using mobile payment are predominantly male and older and have lower education levels, which is similar to the conclusion of Liu J (27).

**Table 3:** Descriptive statistics of variables.

Category	Variable name	Definition	Average/mean value	Standard deviation
Variable type	Buy good new varieties	Whether to buy excellent new varieties, yes = 1, no = 0.	0.839	0.367
		Quantity of new varieties purchased/kg	26.450	89.722
		Unit price/yuan for purchasing excellent new varieties.	32.699	33.991
Core explanatory variables	Mobile payment	In the past 12 months, how did you usually receive and pay? Use direct transfer through bank account or other types of formal financial institution accounts, payment through bank cards, payment through mobile phone WeChat or Alipay, or payment through online banking or online platforms, with at least one value of 1, otherwise the value is 0.	0.419	0.513
Control variables	Gender	Assign 1 for man and 0 for woman.	0.791	0.406
	Years of education	Years of education	6.495	3.377
	Age	Age of household head.	59.233	10.379
	Health condition	Health status: 1 = very good; 2 = good; 3 = fair; 4 = bad; and 5 = very bad.	2. 121	0.930
	Marital status	Marital status, 1 = married; 2 = unmarried; 3 = widowed; 4 = Divorce	1.171	0.626
	Family population	Household size.	4.716	3.577
	Number of family cultivated land blocks	Mu of cultivated land actually cultivated in 2021	7.931	23.007
	Rice yield	In 2021, the yield per mu of rice (if it is wet, it will be converted into dry grain) kg/mu.	5.216	2.926
	Grain storage time	In 2021, the time/month for your family to store grain to maintain family consumption.	10.217	41.851
	Do you use herbicides?	Use = 1, do not use = 0.	0.735	0.441
	Do you apply pesticides?	Yes = 1, No = 0.	0.776	0.416
	Whether to use manual spraying?	Use = l, do not use = 0.	0.790	0.407
	Drought resistance characteristics of varieties	Drought resistance = l, no drought resistance = 0	0.7 l5	0.45 L
	Characteristics of waterlogging resistance of varieties	Waterlogging = l, non-waterlogging = 0	0.632	0.482
	Lodging resistance characteristics of varieties	Lodging resistance = l, lodging resistance = 0, no lodging resistance = 0.	0.642	0.479
	Insect-resistant characteristics of varieties	Insect resistance = l, insect resistance = 0, no insect resistance = 0.	0.577	0.494
	Disease resistance characteristics of varieties	Disease resistance = l, no disease resistance = 0	0.606	0.498

Table 4 shows that the number and unit price of purchasing excellent new varieties of two groups of sample farmers who use mobile payment and those who do not. The results indicate that the number and unit price of purchasing excellent new varieties by farmers who do not use mobile payment are significantly lower than those who use mobile payment, which initially shows that using mobile payment can promote farmers’ behavior of purchasing excellent new varieties.

**Table 4:** Group differences between mobile payment and purchase of excellent new varieties.

Indicator	Unused mobile payment	Use mobile payment	Mean difference
Purchase quantity of excellent new varieties	l7.6l5	46.05	−28.434***
Unit price of purchasing excellent new varieties	38.324	40.482	−2.158*

*, **, and ***, respectively, represent significant at the statistical level of 10, 5, and 1%.

### 4.3 Model setting

To reduce potential selection bias, the Heckman method is used to address self-selection bias in two stages (29). There are three specific cases for selecting the exact specification of the Heckman equation: First, the sample selection is not random (such as incomplete samples due to subjective screening by researchers or lack of data); second, variables are related to error terms (endogenous problems); third, there is bidirectional causality or omitted variable effects that impact the accuracy of the estimates.

The main rationale for choosing the Heckman selection equation in this study is that, theoretically, the behavior of rice farmers purchasing excellent new varieties involves two stages: whether to purchase and the degree of purchase. The degree of purchase of excellent new varieties can only be observed among the rice farmers who have purchased them, while relevant data from those who have not purchased are missing. Therefore, there is sample selection bias when studying the factors influencing the purchase of excellent new varieties, which can lead to biased estimation results. To overcome the above problems, this study uses the Heckman selection equation to analyze the behavior of rice farmers in purchasing excellent new varieties.

Heckman’s two-stage model involves two equations, namely, the selection equation and the outcome equation. The selection equation uses a Probit model to estimate the probability of farmers purchasing excellent new varieties. The independent variable function is as follows:


Prob(D=1∣Z)=φ(Zε) (1)

Where D represents the virtual variable of farmers’ purchase of excellent new varieties (representing the quantity and unit price of farmers’ purchase of excellent new varieties, the unit price of the quantity without purchasing excellent new varieties, the explanatory variable, and the vector of unobserved factors). Then, the results of probability estimation of the model can be used to predict the quantity and unit price of each farmer’s answer to buy excellent new varieties. The residuals from the selection equation are used to construct the inverse Mills ratio. When significant, it indicates that the sample is self-selected. In this case, an additional independent variable needs to be added in the two-stage OLS estimation to correct for sample selection bias. The equation is as follows: D *= 1* indicates the explanatory variable, and D *= 0* is a vector of unobserved factors. Then, the results of the model’s probability estimates can be used to predict each


$${\text{purchase}}_{i}=\beta_{0}+\beta_{1}\;{\text{payment}}_{i}+\beta_{2}x_{i}+\beta_{3}\lambda +\mu$$ (2)

Among them, it is the dependent variable of the model, representing the number and unit price response of the first farmer to the purchase of excellent new varieties. It is the mobile payment status of the farmers and also the core independent variable of this study; it is the coefficient of the mobile payment status and includes control variables for farmers’ characteristics. The demographic characteristics included in this study are gender, years of education, age, health status, marital status, family size, number of cultivated lands, rice yield, grain storage time, whether herbicides are used, whether pesticides are applied, and whether artificial pesticides are used. The characteristics of excellent new varieties include drought resistance, waterlogging resistance, lodging resistance, insect resistance, and disease resistance variables.

## 5 Empirical analysis

### 5.1 Mobile payment and purchasing behavior of excellent new varieties of rice farmers

The estimation results of the Heckman selection model are shown in Table 5. This model includes two equations, with the selection equation used to estimate the probability of rice farmers using mobile payments, while the “result equation” is about the quantity and unit price of purchasing high-quality rice, which corrects for the self-selection problem together with the mobile payment variable, control variables, and the inverse Mills ratio. Among them, mobile payment has a significant impact on the quantity of excellent new varieties purchased, and it is not difficult to find that the inverse Mills ratio in the Heckman regression model passed the significance level test at 1%.

**Table 5:** Heckman regression model.

Variable	Number of excellent new varieties: Heckman regression model (1)		Unit price of excellent new varieties: Heckman regression model (2)	
	Whether to buy seeds?	Number of seeds purchased	Whether to buy seeds?	Unit price of seed purchase
Mobile payment	2.611(0.721)	13.355*(7.353)	1.987***(0.646)	3.077*(1.781)
Gender	−0.059(0.378)	6.944(9.547)	−0.279(0.328)	1.551(2.239)
Years of education	−0.043(0.058)	−0.155(1. 169)	−0.066(0.046)	−0.202(0.275)
Age	0.025(0.016)	0.696*(0.421)	0.026*(0.014)	0.073(0.097)
Health condition	−0.009(0. 187)	−2.707(3.916)	0.139(0. 174)	−0.339(0.910)
Marital status	−0.228(0.329)	−1.995(6. 120)	−0.335(0.293)	0.445(1.428)
Family population	−0.077(0.069)	−1.379(1.710)	−0.162(0. 112)	0.175(0.403)
Number of family cultivated land blocks	−0.003(0.005)	2.870(0. 171)	−0.008(0.006)	−0.091**(0.039)
Rice yield	0.009(0.085)	1.048(1.650)	−0.009(0.082)	0.226(0.390)
Grain storage time	0.004(0.047)	0.232***(0.079)	0.022(0.050)	0.275***(0.018)
Do you use herbicides?	−0.334(0.490)	19.821*(10.593)	0.119(0.529)	−1.039(2.500)
Do you apply pesticides?	1.266(0.674)	49.245***(16. 189)	0.856*(0.492)	0.805(3.820)
Whether to use manual spraying?	0.971(0.706)	−76.133***(16.349)	1.005*(0.555)	3.212(3.850)
Drought resistance characteristics of varieties	9.452(53971.96)	26.772**(11.714)	5.008(164.665)	7.653**(3.393)
Characteristics of waterlogging resistance of varieties	−0.7491(0.616)	−3.730(9.527)	−0.222(0.547)	−3.291(2.236)
Lodging resistance characteristics of varieties	1.116(0.554)	10.031(9.618)	0.612(0.603)	4.008*(2.270)
Insect-resistant characteristics of varieties	−0.869(0.964)	9.178(10.902)	−0.438(0.787)	−4.153(2.552)
Disease resistance characteristics of varieties	1.518(1.011)	−4.478(10.867)	1.115(0.820)	−1.077(2.591)
Inverse Mills ratio (λ)	4.313***(0.033)		2.862***(0.036)	

*, **, and *** mean significant at the statistical level of 10, 5, and 1%, respectively, and the coefficients in brackets are standard errors.

The correlation (*ρ*) is 0.605 in the quantity model of purchasing excellent new varieties and 0.541 in the quantity model of purchasing excellent new varieties, which are consistent with each other.

As can be seen from Table 5, the coefficients corresponding to the inverse Mills ratio are 4.313 and 2.862, both significant at the 1% level, indicating that the aforementioned benchmark model indeed has a sample selection bias issue. After incorporating the inverse Mills ratio, mobile payment remains significant at the 10% level, with regression coefficients of 13.355 and 3.077, indicating that after overcoming the potential sample selection bias, mobile payment can still significantly promote the number and unit price of rice farmers to buy excellent new varieties.

The Heckman regression model (1) shows that mobile payment has passed the significance test for purchasing excellent new varieties at the 10% level, indicating that mobile payment can significantly promote rice farmers’ purchase of excellent new varieties. The possible reasons are as follows: First, it improves transaction efficiency. Traditional agricultural product trading methods often require a long time for manual docking, communication, and transportation. However, through mobile payment tools such as Alipay, WeChat, and mobile banking, farmers can quickly access information on price fluctuations, trading capabilities, and order quantities for new rice varieties online, greatly enhancing transaction efficiency and thus encouraging farmers to use mobile payment tools to purchase excellent new varieties. The dissemination of agricultural information can invisibly increase the family’s attention to agricultural information services and reduce the cost of obtaining agricultural production information, which is consistent with the conclusion of Gabre-Madhin Z E (30). Second, it reduces transaction costs. Farmers use mobile payments, eliminating the need for intermediaries, which removes the profits of middlemen, buyers, and distributors, thereby lowering intermediate transaction costs. Farmers can purchase excellent rice varieties directly from wholesale bases of excellent new varieties, saving on sales channels and transportation costs, which is consistent with the conclusion of Aubert A B (31). Third, it expands the market scope. Through mobile payments, farmers can purchase from wholesale bases of excellent new varieties all over the country, breaking the lower limit for purchasing excellent new varieties and expanding the procurement range, which is consistent with the research conclusion of Jiankui S (32). Fourth, it improves transaction security. Traditional cash transactions have hidden risks that can easily lead to economic disputes. However, mobile payments use encryption technology and security protocols to ensure the safety and reliability of farmers purchasing excellent new varieties, effectively preventing transaction risks and providing traceable transaction records, which is consistent with the conclusions of Dawei F’s research (33).

The Heckman regression model (2) shows that mobile payment has passed the 10% significant test on the unit price of purchasing excellent new varieties, indicating that the more frequently farmers use mobile payment, the greater the likelihood that rice farmers will purchase excellent new varieties. Possible reasons are as follows: First, there is a lack of accountability on mobile payment platforms. Mobile payment platforms employ “inductive design” (such as default opt-in, zero-cost welfare traps, automatic deductions, hidden cancel buttons, and bundled sales), while confidential payments exacerbate operational risks. According to the “Analysis of Complaints Accepted by the National Consumers Association in the First Quarter of 2024” issued by the China Consumers Association, confidential payments and automatic deductions have become the main culprits of induced consumption. In the first 5 months of 2024 alone, the online complaint platform “Black Cat Complaint” received nearly 50,000 complaints related to confidential payments, reflecting the prevalence of confusion surrounding confidential payments. Mobile payments have increased the possibility of online bundling of agricultural products; when purchasing excellent new varieties, it is often necessary to simultaneously purchase agricultural insurance of the same level, and the implicit bundling of mobile payments indirectly raises the unit price of excellent new varieties. According to a survey by the Youth Survey Joint Questionnaire Network, 80.6% of respondents stated that bundling is common, while 20.2% of respondents believe it is very common. The most common phenomenon that respondents encounter in bundling is that they are tied to travel insurance when buying tickets or air tickets (46.5%), followed by hotel or car rental coupons when buying tickets or air tickets (41.8%), and car insurance and beauty services when buying cars (41.5%). Other bundled sale phenomena encountered by the interviewees include online shopping tied to express punctual insurance (35.8%), beauty products tied to related products (30.2%), shopping spree tied to supermarkets (28.6%), downloading apps and installing other software (28.5%), and gas appliances tied to natural gas (25.6%). Second, major banks in China have signed agreements with mobile payment platforms, stipulating that consumers will be charged withdrawal or transfer fees when using mobile express payment services, and these fees will be passed on to consumers by raising product prices, ultimately borne by the consumers (34). Third, rural residents lack financial knowledge and the ability to use mobile payments. Among the sampled farmers, those with 4G/5G phones use their phones for an average of 2.78 h per day. Only 9.37% of the sample farmers said that they had “received computer or mobile phone training” (35). 28.69% of farmers encountered some difficulties in usage, and 28.31% of farmers found it very difficult to use 4G/5G phones, which are only used for making calls. Farmers have a weak ability to use mobile payments, which can easily lead to operational errors, indirectly resulting in an increase in the unit price of purchasing excellent new varieties.

Age has a significant positive impact on the quantity of seeds purchased and the decision to buy high-quality seeds. The possible reasons are that, on the one hand, older farmers generally have more knowledge about the growth, resistance, quality, germination rate, and survival rate of new rice varieties; on the other hand, older farmers often have access to reputable producers and smooth channels for purchasing high-quality rice seeds. Therefore, the understanding of variety characteristics and familiarity with suppliers encourages “rational” older farmers to pursue the optimization of variety quality when purchasing high-quality rice seedlings, which is consistent with Clifton Makate’s research conclusion (36).

The number of family plots has a significant negative impact on the unit price of high-quality seeds at the 5% level. The possible reason is that the more family plots there are, the more severe the fragmentation of land becomes, which increases the production costs of rice cultivation, reduces labor productivity, and suppresses farmers’ demand for purchasing high-quality varieties. As a result, the unit price of excellent rice varieties also decreases, which is consistent with Fang W’s research conclusion (37).

Storage time has a significant positive impact on the quantity and unit price of excellent new varieties, with a significance level of 1%. This indicates that the longer the storage time, the better new varieties farmers purchase, and the higher the unit price of excellent new varieties. The possible reason is that, compared to ordinary rice varieties, excellent rice varieties have characteristics such as fuller grain structure, high yield, stress resistance, disease resistance, and high quality. As long as the temperature difference and humidity are properly controlled, the quality of high-quality rice can be greatly improved. The longer the storage time, the stronger the storage resistance of high-quality rice, making farmers more inclined to purchase excellent new varieties, and the unit price of excellent new varieties also rises with increasing market demand, which is consistent with the conclusion of Afonnikov Dmitry A (38).

The use of herbicides has a significant positive impact on the number of high-quality varieties purchased, with a significant level of 10%. This indicates that the more frequently farmers use herbicides, the better new varieties they purchase. The possible reason is that farmers know how to select appropriate herbicides at different growth stages of the lawn. For example, to effectively reduce the number of weeds before sowing, farmers implement the strategy of “light watering and thin fertilization to promote early germination of weeds.” Farmers also know how to scientifically use herbicides according to recommended dosages, which not only ensures soil sealing and removes weeds but also ensures the vigorous growth of excellent new varieties, thereby increasing the number of excellent new varieties purchased by farmers, which is consistent with Horna’s research conclusion (39).

The use of pesticides has a significant positive impact on the number of high-quality varieties purchased, reaching a level of 1%. This indicates that when farmers use pesticides, the number of new varieties they purchase increases. The possible reason is that in 2020, the variety structure of new pesticides in China continued to optimize, with efficient and low-risk pesticides gradually replacing highly toxic and high-risk ones. The safety and activity of new pesticide products are relatively high, which can reduce the dosage and frequency of application. This not only ensures the goal of precise medication and “zero growth” in pesticide use but also reduces the damage caused by the rice leaf roller during the seedling and tillering stages, lowering the probability of panicle death caused by the rice leaf roller during the jointing stage. It avoids white head losses and damaged plants caused by the rice leaf roller during the heading stage, thereby improving the yield and quality of high-quality rice and increasing the number of farmers purchasing excellent rice varieties, which is consistent with the research conclusion of Zhe C (40).

Using manual spraying has a significant negative impact on the number of high-quality varieties purchased, reaching a level of 1%. This indicates that if farmers use manual spraying of pesticides, the number of new varieties purchased will decrease. The possible reasons are that manual spraying of pesticides is time-consuming and labor-intensive while also consuming water and chemicals. Moreover, the nozzles of the sprayers used for manual spraying are relatively coarse, and the physical and chemical degree of the liquid pesticides sprayed is low, which is not conducive to forming a finished product film, thereby reducing the survival rate of excellent new varieties and further decreasing the number of farmers purchasing excellent new varieties, which is consistent with the research conclusion of Zhe C (40).

The drought resistance characteristics of varieties have a significant positive impact on the quantity and unit price of high-quality varieties purchased, reaching a level of 5%. The possible reason is that water used for rice production accounts for 70% of agricultural water and consumes 50% of China’s total water resources, while drought has become a major reason for the decline in China’s grain production. Therefore, developing drought-resistant, excellent rice varieties can not only reduce non-point source pollution and soil erosion but also ensure water conservation, drought resistance, high yield stability, and high-quality rice. Taking Hanyou 3 as an example, under planting conditions that save more than 50% of water, the yield per mu is as high as 561.6 kg, and its brown rice rate is 79.5%. The milled rice rate is 69.2%, the head rice rate is 52.0%, and the characteristics of high drought-resistant rice yield and high yield per mu promote farmers to buy excellent rice varieties and increase the unit price of excellent rice varieties, which is consistent with the research conclusion of Yuee L (41).

The lodging resistance of varieties has a significant positive impact on the unit price of purchasing high-quality varieties at the level of 10%, indicating that strong lodging resistance of varieties can improve the unit price of high-quality varieties. The possible reason is that lodging of rice during the maturity stage can reduce the yield and quality of rice and increase harvesting costs. Rice varieties with strong lodging resistance can not only reduce the harvesting costs of rice but also increase rice yield, indirectly lowering the production costs of rice cultivation, enhancing farmers’ willingness to purchase high-quality rice seeds, thereby promoting the unit price of high-quality varieties, which is consistent with the conclusion of Xiaoyun L (42).

### 5.2 Robustness test

#### 5.2.1 Replacement sample values

Considering the special physical fitness and health conditions of farmers, this study removed the samples and conducted regression analysis on farmers over 60 years old, excluding the interference of some special factors to make the results more representative. The results are shown in Table 6. Mobile payment still has a significant positive impact on the quantity and unit price of excellent varieties purchased by farmers, further verifying the robustness of the previous results.

**Table 6:** Robustness test.

Variable	Replacement sample values			
	Whether to buy seeds?	Number of seeds purchased	Whether to buy seeds?	Unit price of seed purchase
Mobile payment	2.753***(0.782)	14.929** (7.467)	1.017***(0.242)	17.250*** (3.203)
Control variable	Control	Control	Control	Control
Inverse mills ratio (*λ*)	4.3182***(0.033)		3.591***(0.033)	

Variable	Replacement independent variables			
	Whether to buy seeds?	Number of seeds purchased	Whether to buy seeds?	Unit price of seed purchase
Mobile payment frequency	0.178(0.090)	8.374**(3.752)	0.020***(0.091)	1.824*(1.039)
Control variable	Control	Control	Control	Control
Inverse mills ratio (λ)	4.298***(0.031)		3.014***(0.031)	

Variable	Tail shrinking treatment			
	Whether to buy seeds?	Number of seeds purchased	Whether to buy seeds?	Unit price of seed purchase
Mobile payment	2.573***(0.706)	9.960*(5.757)	2.330***(0.415)	3.920** (1.530)
Control variable	Control	Control	Control	Control
Inverse mills ratio (λ)	4.018(0.032)		2.743***(0.035)	

Variable	Replacement model method			
	Quantitative negative binomial regression		Negative binomial regression of unit price	
Mobile payment	0.773***(0. 169)		0.328***(0.089)	
Control variable	Control	Control	Control	Control
Wald chi2(18)	406.04***		292.69***	
Log pseudolikelihood	–1875.6869		−2388.6469	

*, **, and *** mean significant at the statistical level of 10, 5, and 1%, respectively, and the coefficients in brackets are standard errors.

#### 5.2.2 Replacement independent variables

Using “In the past 12 months, how often have you used mobile payment” as the variable for mobile payment frequency, where 1 = rarely, 2 = occasionally, 3 = sometimes, 4 = often, and 5 = always, and incorporating it into the measurement model as shown below. It can be seen that the measurement results of the mobile payment frequency model are consistent with those in Table 5. The frequency of mobile payment still has a significant positive impact on the number of seeds purchased and the unit price of seed purchased, which further confirms the robustness of previous results.

#### 5.2.3 Tail shrinking treatment

To avoid the influence of extreme values on the regression results, the dependent variables and independent variables were truncated by 1% before regression. The results are shown in Table 6, and the conclusions are the same as those in Table 5, indicating that the estimates in Table 5 are robust.

#### 5.2.4 Propensity score matching method

In this study, the propensity score matching method is used for endogeneity analysis. Table 7 shows that the results of nearest neighbor matching, radius matching, and kernel matching are basically the same, with ATT being significant at the 1, 1, and 5% levels. From the ATT values, if farmers do not use mobile payments, the quantity effect of purchasing excellent new varieties is 13.126 to 25.864; if farmers use mobile payments, the quantity effect of purchasing excellent new varieties is 45.197 to 48.505, with AAT increasing by 22.641 to 32.071, and the average treatment effect being 28.243, indicating that farmers use mobile payments to purchase excellent new varieties.

**Table 7:** Propensity score matching method.

**Quantity**	Processing group	Control group	ATT	Standard deviation	*T* value
Nearest neighbor matching	48.505	18.487	30.018***	9.865	3.04
Radius matching	48.505	13.126	35.379***	9.511	3.72
Kernel matching	45.197	25.864	19.333**	9.772	1.98
Average/mean value	——	——	28.243	——	——

**Price**	Processing group	Control group	ATT	Standard deviation	*T* value
Nearest neighbor matching	38.327	33.894	4.432***	1.733	2.56
Radius matching	38.327	27.451	10.876***	1.887	5.76
Local linear regression matching	37.674	33.342	4.331**	2.465	1.76
Average/mean value	——	——	6.546	——	——

*, **, and *** indicate significant at 10, 5, and 1% levels, respectively.

The results of nearest neighbor matching, radius matching, and local linear regression matching are basically the same, with ATT being significant at the 1, 1, and 5% levels. From the ATT values, if farmers do not use mobile payments, the unit price effect of purchasing excellent new varieties is 27.451 to 33.894; if farmers use mobile payments, the unit price effect of purchasing excellent new varieties is 37.674 to 38.327, with ATT increasing by 4.433 to 10.223, and the average treatment effect being 6.546, indicating that farmers using mobile payments significantly increase the unit price of excellent new varieties purchased.

#### 5.2.5 Replacement model method

Poisson regression assumes that the variance equals the expectation and the data are uniformly distributed, typically used when the dependent variable is a non-negative integer. Negative binomial regression assumes that the variance is significantly greater than the expectation and that the data are over-dispersed. An important assumption of the Poisson regression model is that the conditional mean of the dependent variable equals the conditional variance. If this assumption is not met, the regression results obtained from the Poisson regression model will be biased. It was found that there is a large difference between the mean and variance of the dependent variable; therefore, this study preliminarily suggests that negative binomial regression can be used. Table 6 shows that the use of the negative binomial regression method still has a significant positive impact on farmers’ purchases of excellent varieties in terms of quantity and unit price. This conclusion is consistent with Table 5, where the estimated results in Table 5 are robust. If this assumption does not hold, the regression results obtained from the Poisson regression model will be biased. The survey found a large difference between the mean and variance of the dependent variable, so this study initially suggests that negative binomial regression can be used, as shown in Table 6.

### 5.3 Endogenous test

#### 5.3.1 Balance test

To ensure the estimation quality of propensity score matching, it is necessary to check the balance of the four matching methods to examine whether there are systematic differences in explanatory variables between the treatment group farmers and the control group farmers after matching. The results are shown in Table 8. After matching with the three methods, the Pseudo R2 value is almost zero, and the likelihood ratio of the LR chi-square is significantly rejected at the 1% significance level before the test but not rejected after the test. After matching with the three methods, the Mean Bias and the MedBias standard bias are significantly reduced, so it can be inferred that the dominant bias of observable variables between the treatment group and the control group is reduced to the maximum extent.

**Table 8:** Balance test of matching quality.

Matching method	Pseudo R2	LR chi2	Mean bias	MedBias	*B* value
Before matching	0.172	125.02***	34.0	34.4	103.3*
Nearest neighbor matching	0.063	38.57	13.5	10.5	60.4*
Kernel matching	0.020	12.26	6.6	5.9	33.4*
Local linear regression matching	0.043	26.29	6.8	6.4	49.2*

*, **, and *** indicate significant at 10, 5, and 1% levels, respectively.

#### 5.3.2 Common-support graphs

**Figure 3:**
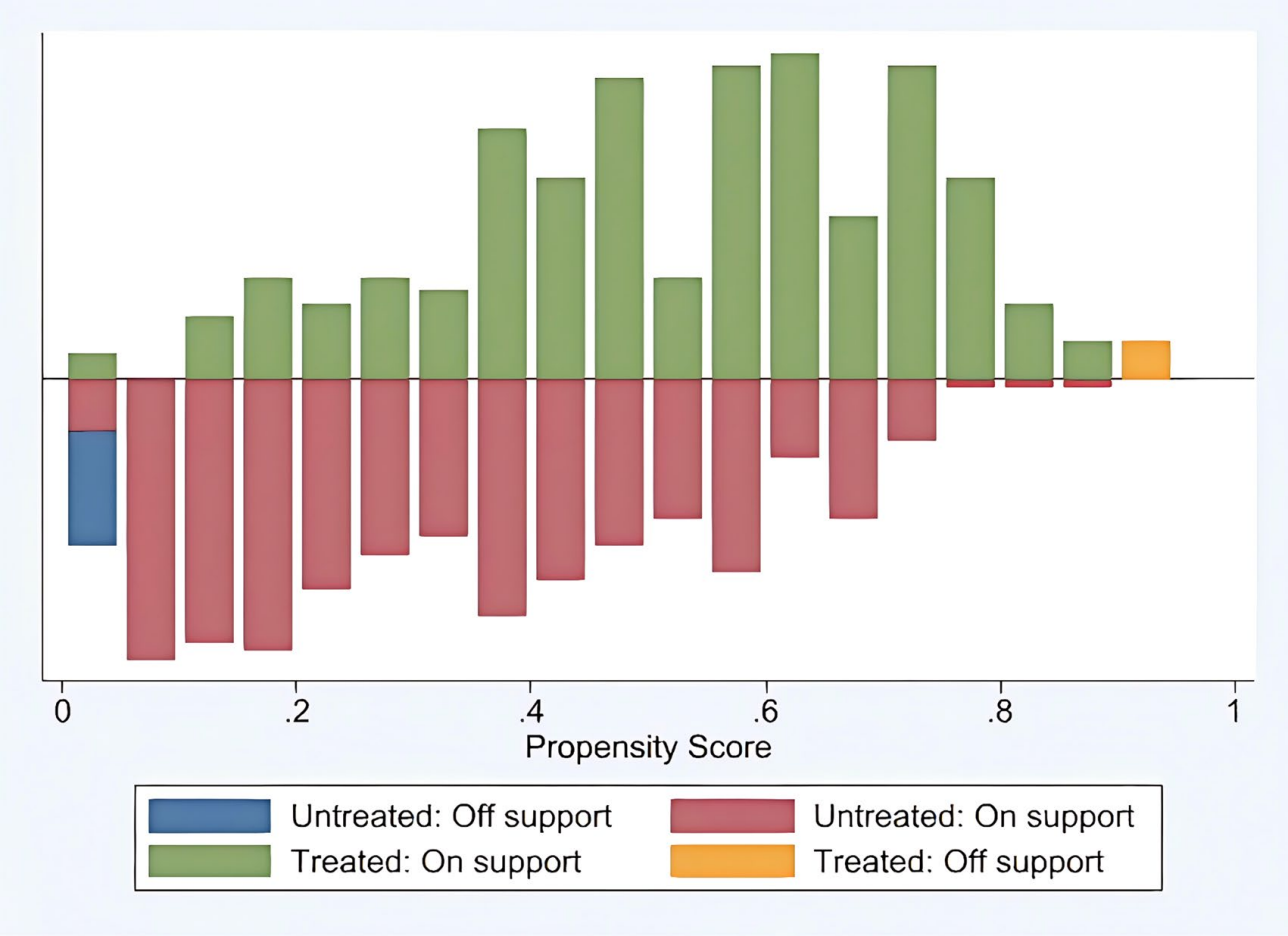
Common-support graph.

The matching results show that among the 317 samples with mobile payment behavior, one did not find a matching object, while among the 219 samples without mobile payment behavior, one also did not find a matching object. After matching using the PSM method, the sample size of the experimental group is 4, and all samples in the control group are within the common support area. In this study, the propensity scores of the original samples in both groups mostly fall within the common support range, indicating a strong degree of matching, as shown in Figure 3.

#### 5.3.3 Sensitivity to the caliper

PSM addresses the issue of selection bias in covariates in non-randomized experiments through matching. However, there still exist confounding variables that are not included in the covariates, which may lead to “hidden deviation,” necessitating further sensitivity analysis of the matching results of the propensity scores. The gamma coefficient (*γ*) is used to represent the influence of undiscovered confounding variables on whether farmers use mobile payments. When γ > 2 and the original PSM results remain significant, the original conclusion can be validated through sensitivity testing. The results of the sensitivity analysis are shown in Table 9. When the γ coefficient increases to 2 in two dependent variables, the original PSM result is still significant at the level of 0.00 L, which shows that the average processing effect results of propensity score matching in two dependent variables are not sensitive to potential confounding variables.

**Table 9:** Sensitivity analysis results of tendency score matching method.

Dependent variable	丫	Sig+	Sig-	t-hat+	t-hat-
Quantity	l	0	0	32.5	32.5
	l.2	0	0	30	34.5
	l.4	0	0	27.5	35
	l.6	0	0	26	37.5
	l.8	0	0	25	37.5
	2	0	0	25	38
Unit price	l	0	0	30	34.5
	l.2	0	0	27.5	35.5
	l.4	0	0	25	37.5
	l.6	0	0	25	37.5
	l.8	0	0	23.5	39.5
	2	0	0	22.5	40

#### 5.3.4 Input propensity score

As shown in Table 10, the covariates selected in this study have a significant impact on the behavior of rice farmers using mobile payments, among which age and marital status have a significant negative effect on rice farmers’ use of mobile payments. In other words, the older the rice farmers are, the lower the probability of using mobile payments. A possible reason is that older rice farmers tend to have lower education levels and are unfamiliar with the operation of mobile payment APP, especially since the elderly often face issues such as small font sizes and complex functions. Unmarried rice farmers have a lower probability of using mobile payments, which may be because they have long been accustomed to cash transactions and find it difficult to change; they tend to prefer cash payments, believing it to be more reliable and not dependent on external devices. The years of education, number of family members, area of farmland, drought-resistant characteristics, and insect-resistant characteristics of the varieties have a significant positive impact on rice farmers’ use of mobile payments, increasing the probability of their use. The probability of using mobile payment is higher for rice farmers with higher education years. A possible reason is that rice farmers with longer years of education generally have a higher acceptance of new technologies and find it easier to master the skills of mobile payment operations. Rice farmers with larger families have a higher probability of using mobile payments, which may be due to the increase in family size directly expanding consumption demand. With the increase in rural residents’ income and the upgrading of consumption, farmers with larger families have higher requirements for payment convenience. Rice farmers with larger areas of farmland have a higher probability of using mobile payments because a large amount of family farmland can lead to large-scale cultivation, expanding the agricultural production input of rice farmers, while mobile payments can meet the comprehensive needs of rice farmers, from daily income and expenditure to agricultural production fund management. The more drought-resistant and insect-resistant rice varieties there are, the higher the probability that farmers will use mobile payments. A possible reason is that genetically modified insect-resistant rice has stronger drought-resistant characteristics due to genetic improvement, while hybrid rice achieves insect-resistant characteristics through genetic engineering, reducing the time farmers spend working in the fields and increasing the possibility of using mobile payments during their leisure time.

**Table 10:** Logit estimation results of covariate entry tendency score.

Indicator name	Coefficient	Standard deviation	*p*-value
Gender	−0.037	0.156	0.810
Years of education	0.037*	0.019	0.057
Age	−0.029***	0.006	0.000
Health condition	−0.063	0.066	0.335
Marital status	−0.228**	0.115	0.048
Family population	0.051*	0.028	0.069
Number of family cultivated land blocks	0.004*	0.002	0.068
Rice yield	0.045	0.027	0.103
Grain storage time	0.001	0.002	0.784
Do you use herbicides?	0.051	0.187	0.783
Do you apply pesticides?	0.308	0.274	0.261
Whether to use manual spraying?	0.062	0.278	0.822
Drought resistance characteristics of varieties	0.754***	0.167	0.000
Characteristics of waterlogging resistance of varieties	−0.058	0.167	0.727
Lodging resistance characteristics of varieties	0.022	0.168	0.894
Insect-resistant characteristics of varieties	0.363*	0.193	0.059
Disease resistance characteristics of varieties	−0.145	0.189	0.442
Log likelihood = −309.839	Pseudo R2 = 0.178	Prob > chi2 = 0.000	

#### 5.3.5 IV method

It is found that mobile payments have a significant positive impact on farmers’ purchase of excellent new varieties, but this result may be affected by endogeneity issues. The first is the reverse causal problem. The quantity and unit price of excellent new varieties purchased can affect farmers’ use of mobile payments, meaning that farmers who purchase more excellent new varieties may have a greater demand for them and tend to use mobile payments to search for information about excellent new varieties, leading to reverse causal problems. An increase in the unit price of excellent new varieties may cause market fluctuations, prompting farmers to use mobile payment technology to understand the price fluctuations of excellent new varieties. Therefore, the possibility of reverse causality cannot be ruled out. The second is the problem of omitted variables. Due to the difficulty of controlling all factors that affect the quantity and unit price of excellent new varieties purchased by farmers, the ability of farmers to accept new things and the variable of family members’ knowledge level, which is difficult to measure accurately, can also affect their purchases of excellent new varieties, so the endogeneity problems caused by omitted variables cannot be ruled out. In addition, there is a certain degree of self-selection issue in the decision-making process regarding farmers’ purchase of excellent new varieties and their use of mobile payments.

Referencing Yin Zhichao et al., whether or not to own a smartphone is considered an IV (8). On the one hand, smartphones are the core carriers of mobile payments, and their hardware (NFC chip and camera) and software (mobile payment application) provide technical basic support for mobile payments. The greater the likelihood of owning a smartphone, the higher the probability of using mobile payments, and the higher the level of mobile payments, the stronger the correlation of the IV; on the other hand, whether farmers own a smartphone does not directly determine the quantity and unit price of excellent varieties purchased, and whether farmers own a smartphone does not change with other factors, satisfying the externality of the IV. The following two auxiliary methods are used to demonstrate this. First, the correlation coefficient between the dependent variable and the IV is directly measured. The results show that the Pearson correlation coefficient between owning a smartphone and purchasing high-quality varieties and unit prices is only 0.023 and −0.003, respectively, while the Spearman correlation coefficients are only −0.078 and −0.053, respectively, indicating a very low degree of correlation. This suggests that there is no correlation between owning a smartphone and purchasing high-quality varieties and unit prices. Second, referring to the research of Ashraf (43), the core explanatory variable is replaced by the IV to regress the explained variable (all control variables are included), with the results shown in Table 11. Furthermore, both the core explanatory variable and IV are included in the model for regression (both control variables are included).

**Table 11:** Estimation results of exogeneity test of tool variables.

Variable	Purchase quantity	Purchase unit price	Purchase quantity	Purchase unit price
Do you have a smartphone?	3.793(7.495)	0.811(2.845)	0.831(7.421)	−0.517(2.800)
Mobile payment			32.444***(7.528)	14.558***(2.841)
Control variable	Have	Have	Have	Have

Table 11 shows that IV has no significant effect on the explained variables. The above results indicate that the IV meets the conditions of relevance and exogeneity, making it a suitable IV.

In addition, the ownership and use of smartphones are two distinct concepts. The ownership of smartphones only affects the seed purchasing channels when relying on payment methods, while the use of smartphones can directly influence seed purchases or farmers’ production activities. Xin Deng (44) pointed out that the use of smartphones significantly promotes farmers’ participation in the recycling of pesticide packaging waste. Tang Lin (45) verified that the use of smartphones indirectly affects farmers’ willingness to participate in centralized treatment of rural domestic sewage through the mediating effects of information search costs, environmental awareness, and social capital. The above research shows that social capital represents information channels and the social functions of smartphones may affect seed purchasing behavior, but this study only studies mobile payment and the purchasing behavior of quality seeds; the use of smartphones in seed purchasing is another research category.

Furthermore, empirical tests were conducted using the two-stage least squares method. The regression results are shown in Table 12. Model (1) displays the endogeneity test results of mobile payment on the purchase of excellent new varieties. It can be seen that Hausman’s endogeneity test results reject the null hypothesis at the 1% significance level, indicating the presence of an endogeneity problem, and Durbin’s test results also reject the null hypothesis at the 1% significance level. Therefore, it can be concluded that the number of excellent new varieties purchased by farmers is endogenous to some extent. The *F*-value estimated in the first stage is 17.61, exceeding the critical value of 10, and the value of the weak tool variable Minimum eigenvalue statistic is 10.085, exceeding the critical value of 6.66 given by Stock-Yogo test at the 15% level (46). Therefore, the IV selected in this study is not a weak IV and can effectively alleviate the endogeneity problem.

**Table 12:** Tool variable method.

Variable	Model (1)			Model (2)		
	OLS	2sls		OLS	2sls	
		First stage	Stage II		First stage	Stage II
Mobile payment	12.657** (5.977)	——	22.028* (12.491)	4.566*** (1.693)		29.424** (14.321)
Instrumental variable	——	0.489*** (0.040)	——	——	0.140*** (0.044)	——
Control variable	Control	Control	Control	Control	Control	Control
Variance ratio	37.37***	17.61***		18.92***	18.02***	
Wald’s *p*-value		692.55***			248.28***	
Minimum eigenvalue statistics		10.085			10.085	
Durbin test *p*-value		7.089***			4.348**	
Wu–Hausman test *p*-value		6.917***			4.220**	
R2	0.565	0.379		0.396	0.217	
Adjust R2	0.549	0.358		0.375	0.191	
Observed value	537	537	537	537	537	

*, **, and *** indicate significant at 10, 5, and 1% levels, respectively, and the coefficients in brackets are standard errors.

Model (2) shows the endogeneity test results of mobile payment for purchasing excellent new varieties. It can be seen that Hausman’s endogeneity test results reject the null hypothesis at the 5% significance level, indicating the presence of an endogeneity problem, while Durbin’s test results also reject the null hypothesis at the 5% significance level. Therefore, it can be concluded that the unit price of excellent new varieties purchased by farmers is endogenous to some extent. The F-value estimated in the first stage is 18.02, exceeding the critical value of 10, and the value of the weak tool variable Minimum eigenvalue statistic is 10.085, exceeding the critical value of 6.66 given by Stock-Yogo test at the 15% level. Therefore, the IV selected in this study is not a weak IV and can effectively alleviate the endogeneity problem. In model (4), the coefficient effect of mobile payment in the second stage of the IV estimation is 29.424, with a significance level of 1%, further indicating that mobile payment is an important influencing factor for farmers purchasing excellent new varieties.

## 6 Mechanism tests of the influence of mobile payment on the purchase of excellent new varieties

According to the theoretical model, the main mechanisms through which mobile payment affects the purchase of excellent new varieties include: first, promoting the market-oriented transformation of agricultural land circulation; second, alleviating the constraining effect of credit constraints on the purchase of excellent new varieties; and third, releasing the consumption potential of rural residents, optimizing their consumption structure, and promoting farmers’ purchases.

### 6.1 Mechanism for promoting the market-oriented transformation of agricultural land circulation

Qiu Tongwei pointed out that the transfer rent is an important manifestation of the market-oriented transformation of the agricultural land transfer market (47). Rational economic agents participate in market competition by maximizing utility or profit; thus, the price connotation reflects supply and demand, competition, and scarcity, serving as an important signal in the market. If farmland transfer is aimed at profit, the rent level gap between acquaintances and non-acquaintances significantly narrows. Therefore, it is necessary to use farmland rent to represent the degree of marketization of farmland circulation. In addition, there is a special phenomenon in China’s farmland transfer market called “human rent,” where transfer rent may be replaced by gifts or moral assistance, rendering the price mechanism ineffective. Based on the above considerations, this study also uses zero rent transfer to measure the marketization level of agricultural land transfer. When the question “what is the payment amount for the land transferred from your family” is used for “zero rent transfer,” if the choice is “0,” the virtual variable zero rent equals 1; otherwise, it equals 0.

The regression results are shown in Table 13. The results in columns (1) and (2) indicate that the interaction between mobile payment and the average rent per mu is significantly positive at the level of 1%, indicating that mobile payment plays a more significant role in promoting farmers to purchase excellent new varieties in terms of obtaining the average rent per mu. The results in columns (3) and (4) indicate that the interaction between mobile payment and zero-rent transfer is significantly negative at the level of 5%, indicating that mobile payment reduces the probability of farmers transferring farmland with “zero rent,” with market-based transactions characterized mainly by monetary exchange replacing “human rent.” This indicates that mobile payment can encourage farmers to transfer farmland for profit and seek suitable trading partners within a larger market radius to maximize income. This result further proves that under the drive of digital information, traditional inner-ring transaction of agricultural land, which is maintained by kinship and geography, is gradually being deconstructed, and the marketization level of agricultural land circulation is gradually improving. Hypothesis 2 is verified.

**Table 13:** Total amount of land investment subsidies obtained.

Variable	Average rent per mu		Zero rent circulation	
	(1) Quantity	(2) Price	(3) Quantity	(4) Price
Mobile payment	11.047**(5.576)	4.627***(1.703)	12.382**(5.963)	4.530***(1.694)
Average rent per mu	0.003***(0.001)	−0.000(0.000)		
Zero rent circulation			9.534(8.210)	1.401(2.333)
Mobile payment × average rent per mu	0.008***(0.001)	0.000(0.000)		
Mobile Payment × Zero Rent Transfer			−33.216**(16.093)	5.539(4.573)
Other control variables	Control	Control	Control	Control
Variance ratio	43. 12***	16.94***	34.09***	17.13***
R2	0.626	0.396	0.569	0.399
Adjust R2	0.611	0.373	0.552	0.375
Observed value	537	537	537	537

*, **, and *** indicate significant at 10, 5, and 1% levels, respectively, and the coefficients in brackets are standard errors.

The missing variables in benchmark regression may lead to biased estimation results. Referring to the methods of Altonji (48) and Oster (49), the degree of model bias arises from the parameter estimation bias indirectly caused by changes in the estimated coefficient values. First, the equation that includes only the core explanatory variables and the explained variables is estimated, and the coefficient estimation value of the core explanatory variables is obtained, assuming it equals; second, all observable control variables are further added for regression, and the estimated coefficient of the core explanatory variables is obtained, assuming it equals *^β^A*; finally, according to the formula $\sigma =|\frac{\beta_{B}}{\beta_{A}-\beta_{B}}|\;$ given by Altonji et al. (48), the error coefficient of missing variables *σ* is calculated. Theoretically, the influence caused by unobservable factors is relatively small, σ〉1. From the benchmark regression in Table 2, it can be seen that without adding control variables, the estimated values of zero rent for quantity and unit price are 8.671 and 3.951, respectively; that is, *β_A_* = 8.671 and 3.951; after adding control variables such as individual characteristics of farmers, land characteristics, family characteristics of farmers, and agricultural production characteristics, the estimated values of zero rent for quantity and unit price are *β_B_* = 12.382 and 4.530. Therefore, the error coefficients of the missing variables *σ* are 3.33 and 7.82, which are also significantly greater than 1. In other words, it is less likely that the estimation results will be biased due to missing variables in the benchmark regression. This study also verifies that mobile payments reduce the probability of farmers transferring farmland with “zero rent” and promote farmers’ behavior of purchasing high-quality rice through mediating effects.

### 6.2 Mechanism for alleviating the constraining effect of credit constraints on the purchase of excellent new varieties

The wealth level for rural families in China is limited, and farmers are denied a series of loans by formal financial institutions. As a result of financial constraints, they are unable to purchase excellent new varieties. WeChat Pay and Alipay service providers have launched microloan functions based on their payment services, allowing farmers to withdraw cash from microloans at any time. At the same time, as a powerful technical support for mobile payments, the Internet serves as an efficient medium for information dissemination, possessing strong social interactivity. Internet technology has improved the speed and depth of information dissemination, alleviating delays in purchasing excellent new varieties caused by information asymmetry. The enhancement of social interaction may also provide families with richer channels for borrowing.

Credit constraints are caused by the quantity distribution on the supply side, the risk distribution on the demand side, and the distribution of transaction costs. Therefore, the supply and demand variables that affect different allocation mechanisms are the decisive factors of credit constraints.

In China, the use of formal loans is based on legal contracts between formal financial institutions and borrowers. Formal loan applications include not only the necessary paperwork but also the time when the application is accepted or rejected. The borrower’s chances of obtaining a loan depend on many uncertain factors, such as the applicant’s financial strength, the guidance of financial policies, and the bureaucratic judgment of formal lenders. The term for formal loans usually exceeds 1 year. For larger formal loans, borrowers need to provide collateral or group insurance to ensure loan repayment. If the borrower fails to repay the debt on time, there is limited room for negotiation, and further delayed payments will lead to court intervention. In this case, a reasonable expectation is that large, commercially oriented borrowers with good prospects for success will become clients of formal loans, while many poor families are considered ineligible. Unlike formal financing in China, mutual assistance loans among friends and relatives provide flexibility for rural residents, with very low transaction costs. Most informal loans are small loans based on verbal commitments. Federe (50) believes that whether farmers can meet their required loan amounts and whether the current interest rate levels are willing to apply for more loans. Baydas (51) believes that formal credit constraints create a “feeling that loans will be rejected,” thus leading to not applying. Petrick (52) thinks that formal credit constraints mean “applications have been rejected.” Boucher (53) thinks that formal credit constraints are when the credit limits obtained by farmers “cannot meet demand,” and the maximum formal loan actually obtained by farmers is less than the loan size they expected. Peng Yan Ling (54) asked “Have you applied for formal or informal financing in the past 3 years?” and “Was your financial request fully approved?” These questions capture farmers’ nominal demand for external credit. If the answer is “not necessary” or “sufficient funds,” it confirms that the family is not credit-constrained; if the answer is “we applied but were rejected” or “we need credit but did not apply,” it confirms that the family is credit-constrained. Zhao (55) thinks that formal credit constraints can be divided into supply-oriented and demand-oriented credit constraints.

**Figure 4:**
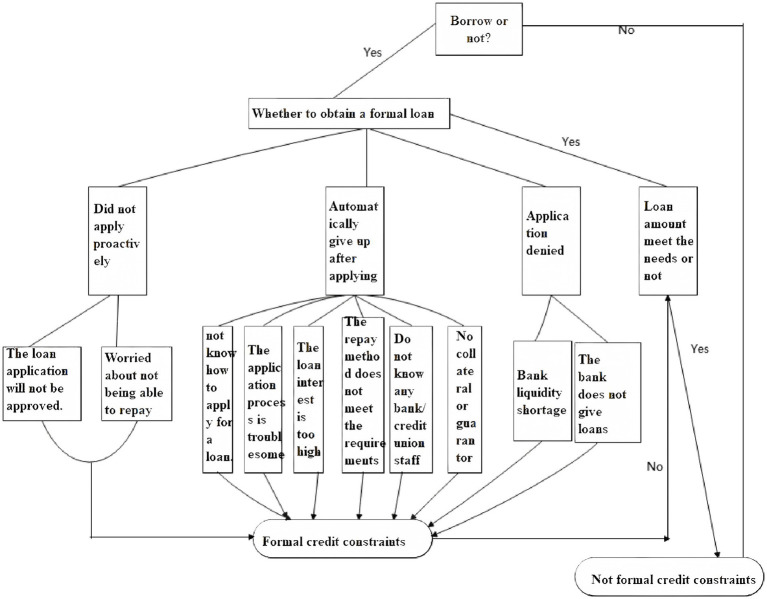
Formal credit constrained a validated index.

In this study, the formal credit constraint variable is “Why has not your family tried to apply for a loan from a bank/credit union to obtain the necessary funds?” The answers to this question include the following: ① The application was rejected; ② I do not know how to apply for a loan; ③ I expect the loan application will not be approved; ④ The application process is troublesome; ⑤ The interest rates on loans are too high; ⑥ The repayment terms or methods do not meet my needs; ⑦ I do not know the staff at bank/credit union; ⑧ There are no mortgages or guarantors. In this study, Option ① and Option ③ are defined as being subject to formal credit. A validated index may be constructed, as shown in Figure 4.

The regression results of formal credit constraints are displayed in column (1) of Table 14. This study introduces the interaction between mobile payment and formal credit constraint through the unit price model for purchasing excellent new varieties and tests whether mobile payment can alleviate the inhibitory effect of formal credit constraints on the unit price of purchasing excellent new varieties. The results indicate that mobile payment plays a more significant role in promoting the unit price of excellent new varieties purchased by farmers under formal credit constraints. This may be because, with the emergence of mobile payment, the channels for farmers’ purchase of excellent new varieties have been enriched, and the strengthening of rural social network connections has also brought many influences, thus alleviating the inhibitory effect of formal credit constraints on the unit price of purchasing excellent new varieties. Hypothesis 3 is verified.

**Table 14:** Ease credit constraints.

Variable	(1) Quantity	(2) Price
Mobile payment	13.162**(6.316)	6.244***(1.666)
Formal credit constraint	−8.888(9.054)	- 16.656***(2.389)
Mobile payment × formal credit constraint	9.801(18.789)	25.504***(4.958)
Other control variables	Control	Control
Variance ratio	33.65***	23.61***
R2	0.566	0.477
Adjust R2	0.549	0.457
Observed value	537	537

*, **, and *** indicate significant at 10, 5, and 1% levels, respectively, and the coefficients in brackets are standard errors.

Frequent use of mobile payment is associated with high personal credit card spending behavior (56), which affects family asset allocation (57) and diversified family investment portfolios (58). Mobile payment platforms can analyze users’ transaction behaviors and credit histories through big data, providing more personalized financial services. For example, the corresponding credit line is provided based on the user’s credit scores. The relaxation of credit constraints will provide strong financial support for rice farmers to purchase high-quality rice, helping them overcome short-term liquidity difficulties, enabling them to make purchases that were previously impossible due to financial constraints, thus unleashing consumption potential.

Digital inclusive finance has significantly improved the consumption experience of rural residents and accelerated the response to consumption demands through a full-process online service. For rice farmers, if they can quickly and conveniently obtain credit support for purchasing seeds through mobile payment platforms, this may directly stimulate their behavior to purchase high-quality varieties. In addition, innovating consumption credit assessment models and utilizing multidimensional data to fill credit gaps will also help break through the bottleneck of rural consumption credit risk control, allowing more rice farmers to obtain credit support.

### 6.3 Mechanism for releasing the consumption potential of rural residents, optimizing their consumption structure, and promoting farmers’ purchases

Consumption decisions are one of the most critical decisions for each family, and consumption expenditure is a core component for measuring family living standards and development potential (59). According to existing research, mobile payment can make consumption more convenient and efficient, providing a platform for families to shop online, which can stimulate the desire for online shopping, change the consumption concepts of rural families, and release their consumption potential. Therefore, this study uses total household consumption expenditure as an indicator to measure consumption potential. The questionnaire inquired in detail about the annual consumption expenditure of the entire family, including food expenditure, clothing expenditure, housing expenditure, durable consumer goods expenditure, daily necessities expenditure, transportation and communication expenditure, cultural leisure and entertainment expenditure, education and training expenditure, medical expenditure, and online shopping expenditure (60). This study summarizes all expenditures involved in the questionnaire to form the total household consumption expenditure. This study uses the Engel’s coefficient and the leisure consumption ratio to measure whether mobile payment can promote the upgrading of the consumption structure. Among them, the Engel’s coefficient is characterized by the proportion of food consumption expenditure to total household consumption expenditure, while the leisure consumption ratio is the proportion of cultural, leisure, and entertainment expenditures to total household consumption expenditure.

Engel’s preliminary research actually discussed the structural changes in residents’ consumption patterns as income increases. This relationship was later described by the Engel curve, which aims to measure the living and welfare levels of residents (61, 62). Engel’s law reveals the structure of consumption and its regularities, holding significant economic meaning, and is regarded by international organizations and academia as one of the main criteria for assessing the wealth level of a country or region. The Engel’s coefficient is the ratio calculated based on Engel’s law, which is the proportion of household food expenditure to total consumption expenditure. The calculation formula is as follows: Engel’s coefficient = Food expenditure/Consumption expenditure ×100%. Engel’s coefficient has become an important indicator for measuring the living standards of residents in the world, and it usually decreases as household income and living standards increase.

The definition of leisure consumption in foreign language studies refers to the consumption of products or services during leisure time, emphasizing leisure activities such as movies, concerts, travel, and sports, which can bring about a good mood and enhance consumers’ happiness (63); in studies conducted in China, the definition similarly refers to leisure activities but also to the consumption expenditures generated during these activities, with the emphasis on leisure expenditure.

Engel’s law indicates that the proportion of food in total expenditure is inversely proportional to family income (or other indicators measuring total family resources). This means that as the Engel’s coefficient for farmers decreases, consumption in other areas increases, thereby raising the probability of purchasing excellent varieties.

Leisure activity consumption has increased the probability of rice farmers purchasing excellent varieties through the following two aspects. First, people can not only engage in part-time agricultural activities during their free time, which brings physical and mental pleasure, but also generate agricultural income; second, leisure activities may also have a social nature. Group activities among rice farmers meet the social interaction needs in leisure activities and provide information and channels for purchasing excellent varieties. Therefore, leisure activities will enhance the effectiveness of people’s purchases of excellent varieties.

**Figure 5:**
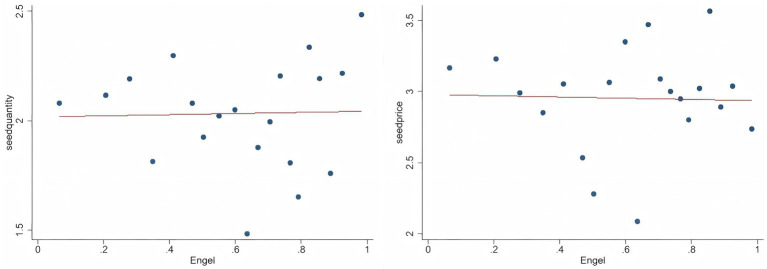
Engel’s coefficient and the quantity and unit price of purchased fine varieties.

**Figure 6:**
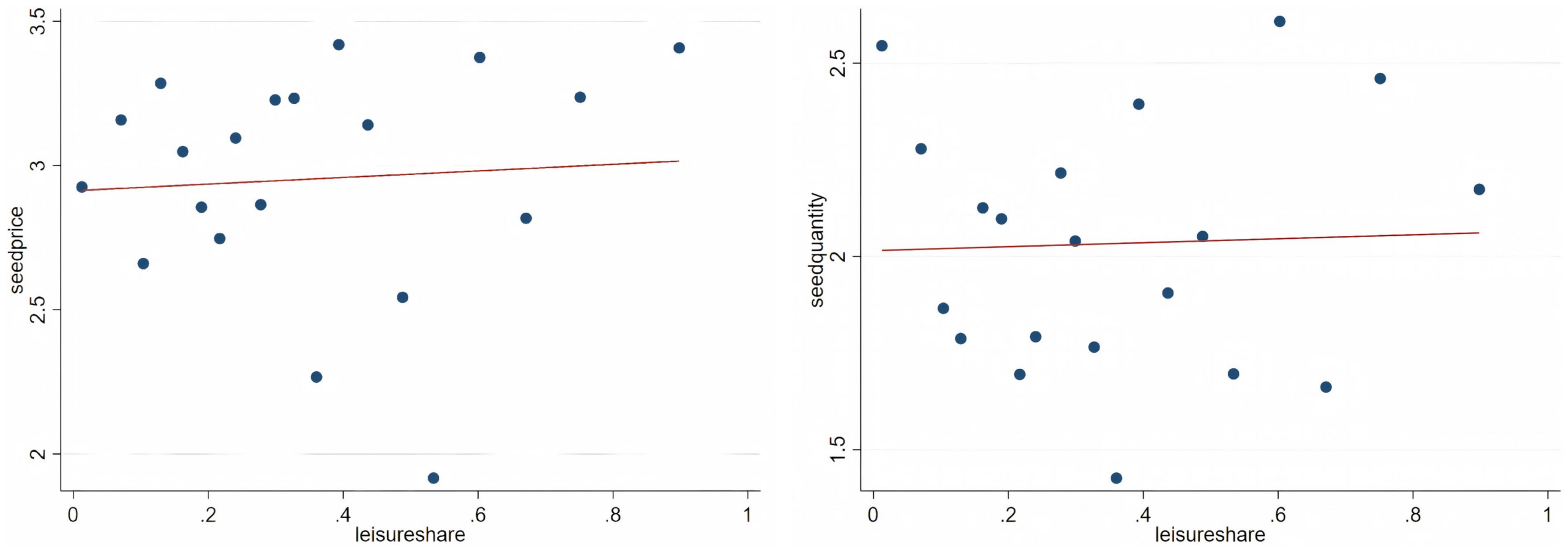
Leisure consumption and purchase of fine varieties quantity and unit price binned scatter.

In this study, leisure consumption, the Engel’s coefficient, as well as the quantity and unit price of excellent varieties are shown in Figures 5, 6. A scatter plot and a fitted trend line were drawn with quantity and unit price as the vertical coordinates and leisure consumption and the Engel’s coefficient as the horizontal coordinates. From Figures 5, 6, it can be seen that Engel’s coefficient has a negative correlation trend with unit price, while it has a positive correlation trend with quantity. Leisure consumption also shows a positive correlation trend with both quantity and unit price. This indicates that Engel’s coefficient and the increase in the unit price for farmers’ purchase of excellent new varieties purchased for leisure consumption by farmers are on the rise, which is basically consistent with the results in Table 15.

**Table 15:** Release the consumption potential of rural families and optimize the upgrading of consumption structure.

Variable	(1) Quantity	(2) Price	(3) Quantity	(4) Price	(5) Quantity	(6) Price
Mobile payment	12.276** (6.058)	5.683*** (1.685)	13.072** (6.016)	4.102** (1.689)	12.662*** (6.015)	3.763*** (1.482)
Total consumption expenditure	0.162 (2.629)	−0.231 (0.731)				
Engel coefficient			2.549 (2.549)	–1.821 (3.446)		
Leisure consumption tendency					−0.619 (12.788)	0.050 (3. 152)
Mobile payment × total consumption expenditure	1.484 (3.613)	4.410*** (1.005)				
Mobile payment × Engel coefficient			1.430 (2.017)	- 1.746*** (0.566)		
Mobile payment × Leisure consumption tendency					0.651 (8.431)	26.726*** (2.078)
Other control variables	Control	Control	Control	Control	Control	Control
Variance ratio	33.52***	18.56***	33.57***	17.79***	33.51***	30.68***
R2	0.565	0.418	0.565	0.408	0.565	0.543
Adjust R2	0.548	0.395	0.548	0.385	0.548	0.525
Observed value	537	537	537	537	537	537

*, **, and *** indicate significant at 10, 5, and 1% levels, respectively, and the coefficients in brackets are standard errors.

The columns (2), (4), and (6) in Table 15 show that the interactions between mobile payment and total consumption expenditure, mobile payment and the Engel’s coefficient, as well as mobile payment and leisure consumption tendency are all significant at the 1% level. This indicates that mobile payment can promote farmers’ behavior of purchasing excellent new varieties by improving their total consumption level, reducing the Engel’s coefficient and improving their leisure consumption tendency. In other words, the reason mobile payment can facilitate farmers’ purchase of excellent new varieties is that using mobile payment helps to tap into consumption potential and optimize consumption structure. Hypothesis 4 is confirmed.

### 6.4 Heterogeneity analysis

Based on the above analysis, the use of mobile payment helps to increase both the quantity and unit price of excellent new varieties purchased by farmers, but this is only an average result. The influence of mobile payment on the quantity and unit price of excellent new varieties purchased by farmers may vary across different groups. Therefore, this study focuses on the heterogeneity of the influence of mobile payment on the quantity and unit price of excellent new varieties purchased by farmers from eight aspects: gender, age, education level, drought resistance, waterlogging resistance, lodging resistance, insect resistance, and disease resistance.

#### 6.4.1 Gender heterogeneity analysis

As can be seen from Table 16, mobile payment has a significant positive impact on the quantity and unit price of excellent new varieties purchased by men, but it has no significant impact on the quantity and unit price of excellent new varieties purchased by women. The possible reason is that most rural families in China follow traditional social divisions of labor, where men are required to participate in agricultural production activities to obtain higher economic income, and the frequency of purchasing agricultural production materials is much higher than that of women. Mobile payment is a convenient payment tool that provides strong support for women to purchase seeds more easily, so men tend to purchase more excellent new varieties and are willing to pay a higher unit price for them (64).

**Table 16:** Heterogeneity analysis.

Heterogeneity type	Gender heterogeneity			
	Man		Woman	
	Quantity	Price	Quantity	Price
Mobile payment	12.831*(7.251)	4.090**(1.967)	5.775(4.527)	0.454(3.539)
Control variable	Control	Control	Control	Control
Variance ratio	32.31***	14.97***	31. 12***	6.50***
R2	0.575	0.385	0.847	0.537
Adjust R2	0.557	0.359	0.820	0.454

Heterogeneity type	Age heterogeneity			
	Quantity	Price	Quantity	Price
Mobile payment	14.331**(6.089)	3.229(3.281)	−0.094(7.551)	4.152**(1.660)
Control variable	Control	Control	Control	Control
Variance ratio	84.58***	4.00***	17.20***	28. 11***
R2	0.858	0.222	0.526	0.645
Adjust R2	0.847	0.166	0.495	0.622

Heterogeneity type	Heterogeneity of education level					
	Primary school		Junior school		Senior high school	
	Quantity	Price	Quantity	Price	Quantity	Price
Mobile payment	4.712(8. 169)	7.080(4.786)	11.266(9.332)	1.816(2.354)	−0.792(2.051)	9.802**(4.33 1)
Control variable	Control	Control	Control	Control	Control	Control
Variance ratio	15.81***	6.89***	33.01***	12.50***	114.89***	9.08***
R2	0.511	0.313	0.767	0.555	0.972	0.733
Adjust R2	0.478	0.267	0.744	0.511	0.963	0.652
Observed value	275	275	188	188	74	74

Heterogeneity type	Drought-resistant heterogeneity			
	Combat drought		Not drought-resistant	
	Quantity	Price	Quantity	Price
Mobile payment	8.511***(2.750)	8.354(5.986)	5.124(4.627)	1.338(1.498)
Control variable	Control	Control	Control	Control
Variance ratio	8.47***	5. 11***	119.58***	8.97***
R2	0.514	0.389	0.847	0.294
Adjust R2	0.453	0.313	0.840	0.261
Observed value	154	154	383	383

Heterogeneity type	Heterogeneity of waterlogging resistance			
	Kanglao		Do not carry waterlogging	
	Quantity	Price	Quantity	Price
Mobile payment	7.865(7.725)	3.615*(2. 145)	11.034(8.697)	2.179(2.863)
Control variable	Control	Control	Control	Control
Variance ratio	26.65***	5.55***	23.39***	19.16***
R2	0.590	0.230	0.680	0.635
Adjust R2	0.568	0.189	0.651	0.602
Observed value	332	332	205	205

Heterogeneity type	Lodging resistance heterogeneity			
	Anti-fall		Do not resist falling.	
	Quantity	Price	Quantity	Price
Mobile payment	9.371(7.915)	6.254***(2.09 8)	5.596(7.829)	4.194(3.058)
Control variable	Control	Control	Control	Control
Variance ratio	25.90***	2.21***	26.66***	22.46***
R2	0.581	0.105	0.711	0.674
Adjust R2	0.559	0.057	0.684	0.644
Observed value	335	335	202	202

Heterogeneity type	Insect resistance heterogeneity			
	Insect resistance		Not resistant to insect lodging	
	Quantity	Price	Quantity	Price
Mobile payment	15.922*(9.218)	2.431(1.797)	−4.249(5.292)	4.834(3.380)
Control variable	Control	Control	Control	Control
Variance ratio	20.67***	2.25***	43.87***	15.45***
R2	0.561	0. 122	0.766	0.536
Adjust R2	0.534	0.068	0.749	0.501
Observed value	292	292	245	245

Heterogeneity type	Resistance to lodging heterogeneity			
	Disease resistance		Not resistant to disease and lodging	
	Quantity	Price	Quantity	Price
Mobile payment	14.585*(8.641)	2.397(2.284)	−3.075(5.768)	2.048(2.527)
Control variable	Control	Control	Control	Control
Variance ratio	20.02***	1.64*	61.13***	29.88***
R2	0.538	0.087	0.832	0.708
Adjust R2	0.511	0.034	0.818	0.684
Observed value	310	310	227	227

*, **, and *** indicate significant at 10, 5, and 1% levels, respectively, and the coefficients in brackets are standard errors.

#### 6.4.2 Age heterogeneity analysis

In this study, samples under 60 years old represent young people, while samples over 60 years old are classified as elderly. As can be seen from Table 10, mobile payment has a significant positive impact on the quantity of excellent new varieties purchased by the elderly. The possible reason is that elderly people in rural China have rich planting experience and are familiar with the growth characteristics of excellent new varieties, such as high growth, thick stems, long ears, and delicate taste. Driven by the dual factors of meeting food self-sufficiency needs and market profit incentives, elderly people use mobile payment to strive to purchase excellent new varieties to meet their own needs.

Mobile payment has a significant positive impact on the prices of excellent new varieties purchased by young and middle-aged people. The possible reason is that young and middle-aged people belong to a group with a high penetration rate of mobile payment, and their income is relatively abundant, making them less sensitive to the prices of excellent rice varieties. The price increase of excellent new varieties has little effect on the price perception of young and middle-aged people, while the convenient and fast characteristics of mobile payment further promote the willingness to purchase excellent new varieties, thereby increasing the price of excellent new varieties purchased (65).

#### 6.4.3 Educational level heterogeneity analysis

From the perspective of education level, Table 16 shows that mobile payment has a significant positive impact on farmers with a high school education or above. The possible reason is that highly educated farmers not only mean that they can use mobile terminal functions to focus on basic aspects such as payment and social interaction but also utilize these functions for richer activities such as shopping, learning, and entertainment. Moreover, families of highly educated farmers imply higher income levels, so the impact of price increases for excellent new varieties is relatively small for them. They are very familiar with the mobile payment method for excellent new varieties, thus increasing the unit price they are willing to pay for purchasing excellent new varieties.

#### 6.4.4 Heterogeneity analysis of drought resistance of varieties

From the perspective of drought resistance of varieties, Table 16 shows that mobile payment has a significant positive impact on the purchase quantity of excellent seeds of drought-resistant varieties. The possible reason is that using mobile payment tools can provide information about the drought resistance of rice varieties, enrich drought-resistant varieties, save water, enhance knowledge levels of yield per mu, and break down information constraints, thereby increasing the purchase quantity of excellent seeds of drought-resistant varieties (66).

#### 6.4.5 Heterogeneity analysis of waterlogging resistance of varieties

From the perspective of waterlogging resistance of varieties, Table 16 shows that mobile payment has a significant positive impact on the unit price of excellent seeds of waterlogging-resistant varieties. The possible reason is that mobile payment products can link to the service mode of Huinong APP, allowing farmers to obtain knowledge about “waterlogging-resistant rice” through the service of benefiting farmers, clarifying that “waterlogging-resistant rice” can respond quickly when floods occur, with stems growing several meters high and developing hollow “breathing tubes,” thus preventing the rice from “drowning” in water.

#### 6.4.6 Analysis of lodging resistance heterogeneity of varieties

From the perspective of lodging resistance of varieties, Table 16 shows that mobile payment has a significant positive impact on the unit price of excellent seeds of lodging-resistant varieties. The possible reason is that farmers can obtain information about the lodging resistance through the use of mobile payment tools, such as scientific fertilization, scientific irrigation, disease prevention and pest control, and increasing silicon fertilizer application. The accuracy of information acquisition encourages farmers to tend to purchase excellent seeds of lodging-resistant varieties, thereby promoting an increase in the unit price of excellent lodging-resistant seeds (67).

#### 6.4.7 Heterogeneity analysis of insect resistance of varieties

From the perspective of insect resistance of varieties, Table 16 shows that mobile payment has a significant positive impact on the purchase quantity of excellent seeds of insect-resistant lodging varieties. The possible reason is that farmers can improve the information about insect-resistant lodging by obtaining six methods of rice disease pest control through mobile payment tools, such as adjusting rice planting density, using insect resistant varieties, biological control, using reasonable pesticides, regular inspection and irrigation management, and the convenience of information acquisition promotes farmers to tend to buy the quantity of excellent seeds of insect-resistant lodging varieties (68).

#### 6.4.8 Heterogeneity analysis of resistance to disease and lodging of varieties

From the perspective of varieties’ resistance to lodging, Table 16 shows that mobile payment has a significant positive impact on the purchase quantity of excellent seeds of varieties with resistance to lodging. The possible reason is that the three major diseases of rice, namely, rice blast, sheath blight, and false panicle disease, are among the main obstacles to high and stable rice yields. If not controlled properly, rice can lose 20–30% per mu, and in severe cases, it may lead to total crop failure. Therefore, mobile payment can promote the occurrence characteristics of major rice diseases and effective control methods, allowing farmers to obtain comprehensive control prevention information through the information service function of mobile payment, which plays a great role in ensuring increased rice production and harvest, thus increasing the purchase quantity of excellent seeds of disease-resistant and lodging-resistant varieties (68).

### 6.5 Research limitations

Influenced by external factors such as “financial service policies” and “seed market norms” as well as other unobservable factors, there may be a reverse causality issue between mobile payment energy and the behavior of rice farmers purchasing high-quality varieties. This may also be due to the limitations of the cross-sectional data used in this study. In the future, we can use time series data and panel data to further study the relationship between mobile payment and the purchasing behavior of rice farmers regarding high-quality varieties and address the issue of causal reversal between them.

## 7 Discussion

Under the background that planting efficiency is improved, food safety is ensured and sales are promoted via digital means. Based on survey data from 617 micro-farmers in Jiangxi Province, this study empirically analyzes the influence of mobile payment on the purchasing behavior of excellent new varieties of rice farmers. First of all, the research results indicate that mobile payment has a significant positive impact on the purchase quantity and unit price of excellent new varieties of rice farmers, which is consistent with the research conclusion of Jimson Nyambu Mwikamba (69) that mobile payment has a significant positive impact on the promotion and sales of new agricultural varieties. In addition, Alejandro Estefan (10) also reached a similar conclusion, proving that digital technology can facilitate the promotion and sales of new agricultural varieties. However, unlike previous studies, this study examines the purchasing behavior of farmers’ fine varieties through mobile payment and characterizes the purchase quantity and unit price of farmers’ fine varieties based on purchase scale and level. The reason is that the scale and level of procurement can reflect the supply and demand relationship, resource allocation efficiency, and economic behavior characteristics of excellent rice variety market. Therefore, shifting the research perspective from the promotion of new agricultural varieties to the purchasing behavior of excellent varieties helps to promote the dynamic relationship between price and supply in new variety technologies and the supply law of agricultural products.

In exploring the impact mechanism, this study analyzes the influence mechanism of mobile payment on rice farmers’ purchase of excellent varieties from multiple angles, including raising the level of land rent, reducing the probability of “zero rent” transfer of land, alleviating formal credit constraints, releasing the consumption potential of rural residents, and optimizing their consumption structure. This is similar to the article (32) that discusses transaction costs and information network acquisition. However, unlike most articles that directly discuss mobile payment and farmers’ purchase of excellent varieties (70), this study verifies the role of three factors, such as land factors, capital factors, and consumption upgrading, in the relationship between mobile payment and farmers’ purchase of excellent varieties, thereby bridging the gap between mobile payment and farmers’ land factor allocation, alleviating financial constraints, and upgrading of consumption. In addition, this study further explores the group gap effect of digital purchasing power among farmers of different genders, education levels, and ages, finding that this group gap effect is more pronounced among men, those with a high school education, and the elderly, which is consistent with the conclusion of Romit Olakiya (12).

Finally, this study analyzes the role of mobile payments in promoting rice farmers’ purchase of excellent new varieties, focusing on characteristics such as drought resistance, waterlogging resistance, lodging resistance, insect lodging resistance, and disease lodging resistance. It finds that mobile payments have a more significant promoting effect in the cultivation of varieties with drought resistance, waterlogging resistance, lodging resistance, insect lodging resistance, and disease lodging resistance. This is consistent with the research findings of Sisay Diriba Lemessa (71). The development of Internet information technology can provide information retrieval channels for the research and development of high-quality new seeds. The government and major research institutions should properly preserve high-quality germplasm resources, establish a sharing platform for high-quality germplasm resources, strengthen the collection and preservation of rare high-quality germplasm resources, emphasize the evaluation and identification of high-quality germplasm resources, provide a genetic basis for cultivation, and timely publish information on an adaptive new varieties on Internet information planning platforms.

## 8 Conclusion and policy recommendations

### 8.1 Conclusion

Based on the scale and level of rice farmers’ purchasing new rice varieties, this study utilizes microdata from 100 villages and 1,000 households in Jiangxi Province to construct the Heckman two-stage model, IVs, and propensity score matching methods to explore the impact mechanism and heterogeneity of mobile payment on rice farmers’ purchasing behavior of new rice varieties. For the above analysis, the following four conclusions can be drawn.

1. Mobile payment can significantly promote the scale and level of rice farmers’ purchase of excellent new varieties, and it has passed the test of endogeneity and robustness.
2. Mobile payment can improve the level of farmland rent by promoting farmland circulation, reduce the probability of “zero rent” farmland circulation, confirm the market-oriented transformation of farmland circulation, ease formal credit constraints, release the consumption potential of rural residents, optimize the mechanism of its consumption structure, and promote rice farmers to purchase excellent new varieties.
3. Mobile payment has a significant heterogeneity in its effect on rice farmers’ purchase of excellent new varieties. The group effect of mobile payment on rice farmers’ purchase of excellent new varieties is more pronounced among men and those with a high school education, while this promoting effect is more evident in the purchasing scale of the elderly and also more obvious in the purchasing level of the youth.
4. From the analysis of the characteristics of excellent varieties, the effect of promoting mobile payments for rice farmers to purchase excellent new varieties is more obvious in the breeding of varieties with drought resistance, waterlogging resistance, lodging resistance, insect resistance, and disease resistance.

### 8.2 Policy recommendations

The conclusions of this study have important policy implications. As a new mobile payment method, it has enormous development potential in the rural market of China. Therefore, it is necessary to promote the popularization and application of mobile payment in rural areas.

1. Optimize the rural payment environment and give full play to the inclusive function of mobile payment. We should support the development of mobile payments in rural areas, improve the rural popularization of planned mobile network facilities and coverage, improve the popularization of mobile phone penetration rate, increase the subsidies of operators, effectively increase the number of smart phone users in rural families, and give preferential treatment to farmers in technology and policies, such as simplifying account opening and quota requirements in policies and issuing subsidies to farmers through mobile payment accounts, thereby improving the acceptance of mobile payments among rural families and further enhancing the role of mobile payments in purchasing and lending.
2. Promote research on rice planting technology, variety improvement, and large-scale production equipment, and guiding farmers to cultivate high-quality rice varieties (72). With technological advancement as the fundamental driving force, we should modernize rice production, strengthen research on cutting-edge technologies for rice planting techniques, variety improvement, and large-scale production equipment, improve the transformation rate of scientific and technological achievements through demonstration and guidance, promote the technology of village-type varieties, and fully utilize technology to support increased food production (73). We will intensify the promotion of excellent grain varieties and key measures for yield increase, guiding farmers to adopt high-quality rice varieties and striving to improve both the yield and quality of rice.
3. In the short term, to play the role of mobile payment in promoting the market-oriented transformation of agricultural land transfer, we can embed websites or services related to agricultural land transfer into widely used digital financial applications, such as WeChat and Alipay, or in locally well-known digital credit or digital insurance platforms, and timely publish and disseminate relevant information about agricultural land transfer on these platforms (74).
4. In the long run, efforts should be made to address the credit constraints faced by farmers in purchasing high-quality varieties, enrich financing channels, support the development of Internet finance, strengthen the supervision of peer-to-peer lending platforms, and provide better financial support for rice farmers to purchase high-quality varieties (75).
5. With the support of mobile payments, actively promote the expansion of new consumption and new business models into rural areas, strengthen the supply-side reform of rural household consumption upgrades, support the development and enjoyment of electronic digital, cultural, educational, entertainment, medical, and healthcare products, and effectively increase the supply of high-quality goods for the new countryside and “new farmers (76).”

## Data Availability

The original contributions presented in the study are included in the article/supplementary material, further inquiries can be directed to the corresponding author/s.
